# Establishing the ELIXIR Microbiome Community

**DOI:** 10.12688/f1000research.144515.2

**Published:** 2025-09-08

**Authors:** Robert D. Finn, Bachir Balech, Josephine Burgin, Physilia Chua, Erwan Corre, Cymon J. Cox, Claudio Donati, Vitor Martins dos Santos, Bruno Fosso, John Hancock, Katharina F. Heil, Naveed Ishaque, Varsha Kale, Benoit J. Kunath, Claudine Médigue, Teresa Nogueira, Evangelos Pafilis, Graziano Pesole, Lorna Richardson, Monica Santamaria, Nikolaos Strepis, Tim Van Den Bossche, Juan Antonio Vizcaíno, Haris Zafeiropoulos, Nils P. Willassen, Eric Pelletier, Bérénice Batut

**Affiliations:** 1 European Bioinformatics Institute, European Molecular Biology Laboratory, Hinxton, UK; 2 Institute of Biomembranes, Bioenergetics and Molecular Biotechnologies, Bari, Italy; 3 ELIXIR Hub, Hixton, UK; 4 Station Biologique de Roscoff, CNRS/Sorbonne Université, Roscoff, France; 5 Centro de Ciências do Mar, Universidade do Algarve, Faro, Portugal; 6 Edmund Mach Foundation Research and Innovation Centre, San Michele all'Adige, Trentino-South Tyrol, Italy; 7 Systems and Synthetic Biology, Wageningen University & Research, Wageningen, Gelderland, The Netherlands; 8 Department of Biosciences, Biotechnologies and Biopharmaceutics, University of Bari, Bari, Italy; 9 Faculty of Medicine, University of Ljubljana, Ljubljana, Slovenia; 10 Berlin Institute of Health Charité, Universitätsmedizin Berlin, Berlin, Germany; 11 Luxembourg Centre for Systems Biomedicine, University of Luxembourg, Esch-sur-Alzette, Luxembourg; 12 Metabolic Genomics, Genoscope, Institut François-Jacob / CEA / CNRS / Université Evry / Université Paris-Saclay, Evry, France; 13 IFB-core, Institut Français de Bioinformatique (IFB), CNRS, INSERM, INRAE, CEA, 94800, Villejuif, France; 14 INIAV—National Institute for Agrarian and Veterinary Research, 4485-655, Vairão, Portugal; 15 CE3c - Centre for Ecology, Evolution and Environmental Changes & CHANGE - Global Change and Sustainability Institute, Faculdade de Ciências da Universidade de Lisboa, 1749-016, Lisboa, Portugal; 16 Institute of Marine Biology, Biotechnology and Aquaculture, Hellenic Centre for Marine Research, Heraklion, Greece; 17 Department of Soil, Plant and Food Sciences (Di.S.S.P.A.), University of Bari, Bari, Italy; 18 Department of Pathology and Clinical Bioinformatics, Erasmus MC Cancer Institute, Erasmus MC, Rotterdam, The Netherlands; 19 Department of Biomolecular Medicine, Faculty of Medicine and Health Sciences, Ghent, Belgium; 20 VIB, UGent Center for Medical Biotechnology, Ghent, Belgium; 21 UiT The Arctic University of Norway, Tromsø, Norway; 22 Research Federation for the study of Global Ocean Systems Ecology and Evolution, CNRS, Paris, France; 23 Bioinformatics Group, Department of Computer Science, Albert-Ludwigs-University Freiburg, Freiburg, Germany

**Keywords:** Microbiome, ELIXIR Community, White Paper

## Abstract

Microbiome research has grown substantially over the past decade in terms of the range of biomes sampled, identified taxa, and the volume of data derived from the samples. In particular, experimental approaches such as metagenomics, metabarcoding, metatranscriptomics and metaproteomics have provided profound insights into the vast, hitherto unknown, microbial biodiversity. The ELIXIR Marine Metagenomics Community, initiated amongst researchers focusing on marine microbiomes, has concentrated on promoting standards around microbiome-derived sequence analysis, as well as understanding the gaps in methods and reference databases, and identifying solutions to the computational overheads of performing such analyses. Nevertheless, the methods used and the challenges faced are not confined to marine microbiome studies, but are broadly applicable to other biomes. Thus, expanding this Marine Metagenomics Community to a more inclusive ELIXIR Microbiome Community will enable it to encompass a broader range of biomes and link expertise across ‘omics technologies. Furthermore, engaging with a large number of researchers will improve the efficiency and sustainability of bioinformatics infrastructure and resources for microbiome research (standards, data, tools, workflows, training), which will enable a deeper understanding of the function and taxonomic composition of the different microbial communities.

## 1. Introduction

The term “microbiome” is a description of an entire habitat that encompasses all the microbes (bacteria, archaea, eukaryotes, and viruses), their composition (genomes, proteins and various molecules they produce), and the environment they are found in. ^1^ The microbiome is experimentally characterised by the application of one or more ‘omics techniques, especially metabarcoding, metagenomics and metatranscriptomics, but also metaproteomics and metabolomics, combined with contextual metadata about the surrounding environment, be it a geographic location (e.g. ocean), host-associated (e.g. human gut) or engineered (e.g. wastewater treatment plant). Over the past decade, scientists have become increasingly aware of the role performed by microbes in the health (or maintenance) of the environment, and that dysbiosis of the microbial community can lead to dysregulation and/or negative outcomes. Furthermore, there can be complex compositional modulation of microbiomes. For example, viruses that infect bacteria are found ubiquitously in all environments and play critical roles in community dynamics. Microbial communities can be very diverse and heterogeneous in composition across geospatial and temporal scales, and the culture-independent methods for identifying species with the microbiome often reveal hitherto unknown microbes. Despite methodological difficulties, understanding the taxonomic and functional composition of a microbiome, how compositional differences relate to phenotypes, and how these communities may be manipulated to restore a community close to a natural composition are key current research questions. Given that most academic institutions have access to dedicated sequencing facilities or equivalent commercial facilities, coupled with diminishing costs of DNA sequencing and other ‘omics technologies, it is relatively easy to generate large datasets. Therefore, there are now millions of microbiome-derived sequence datasets, many of which are large (gigabytes to terabytes) and complex (thousands of related and/or diverse samples), but it can require significant computational resources to store and analyse the data. Additionally, datasets from other ‘omics techniques such as metaproteomics and metabolomics are being increasingly generated, alone or in combination with metagenomics and/or metatranscriptomics data coming from the same samples. A key challenge facing the microbiome research community is how to: appropriately store the data; informatically process, integrate, compare and interpret microbiome-derived data; and how to make the data findable, accessible, interoperable and reusable, i.e. FAIR. ^2^

Such steps are vital to ensure reuse of data and scientific reproducibility. For example, when wishing to contextualise the results between similar experiments, the way a dataset has been produced and processed must be transparent to establish whether it is comparable (e.g. amplified sequence variants (ASVs) can only be compared to those with the same amplified region). Similarly, ensuring that taxonomic and functional assertions are placed in the context of the original sample/sequencing effort and associated contextual metadata is crucial for understanding compositional microbiome changes, such as those in health and disease or longitudinal datasets. Ensuring data is FAIR is typically necessary to comply with most scientific funding sources open access data requirements. Finally, describing how a dataset has been produced allows verification and replication of scientific experiments.

ELIXIR ^3^ is a distributed infrastructure bringing together experts from across Europe to enable life science researchers throughout the world to access and analyse life science data. ELIXIR is formed by member states each with a national Node composed of one or more centres of excellence in bioinformatics. Each Node coordinates services, standards and resources, and collaborates with experts in other Nodes to create a sustainable Europe-wide infrastructure for biological data. ELIXIR Platforms bring together experts from Nodes to develop ELIXIR’s vision and coordinate activities in defined areas. The five Platforms are Data, Tools, Interoperability, Compute and Training. ELIXIR Communities bring together experts across ELIXIR Nodes and external partners to coordinate activities within specific life science domains. During the establishment of ELIXIR, the ELIXIR Marine Metagenomics Community acted as a biome-specific network of researchers for the identification and organisation of domain-specific reference resources, development of reproducible workflows and the proposal of best practices. However, there is no underlying reason to restrict these activities to just the marine environment, with most of the aforementioned efforts broadly applicable to analysis of microbiome-derived sequence data from any biome. Furthermore, there is the need to extend the activities of the Marine Metagenomics Community to integrate expertise and knowledge about other ‘omics technologies, such as metatranscriptomics, metaproteomics and metabolomics, which are increasingly used in microbiome studies. Thus, this white paper outlines some of the historical aspects of the Marine Metagenomics Community and the aims of the broader community, especially in the context of the other ELIXIR Communities and infrastructure platforms.

## 2. From marine metagenomics to a more inclusive Community

The ELIXIR Marine Metagenomics Community, established in 2015 as part of the European Commission funded ELIXIR EXCELERATE project (grant number 676559), was one of the first four ELIXIR Communities created as “Use Cases”. ^4^ During the EXCELERATE project, these ELIXIR “Use Cases” were expanded and renamed to Communities, with a unified aim of bringing European specialists together to provide sustainable data resources, benchmark different tools and workflows, provide access to computing and storage, improve interoperability, and develop training resources within their research domains. These activities were conducted in collaboration with the ELIXIR Platforms, to ensure harmonisation of the outputs. As such, the Marine Metagenomics Community focused on metagenomics analysis pipelines, addressing the lack of reference databases and promoting the best practices for the research community. Highlights include the incorporation of new tools and resources into the MGnify ^5^ and MetaPIPE ^6^ analytical pipelines (e.g. MAPseq, ^7^ ITSOneDB ^8^), the formal description of the MGnify pipeline using the common workflow language (CWL ^9^) to promote interoperability, the establishment of marine metagenomics data (e.g. Marine Metagenomics Portal, MARdb, ^10^ METDB, ^11^ and the Ocean Gene Atlas ^12^) and a community paper (beyond ELIXIR) promoting best practices advocating the use of community standards for contextual provenance and metadata at all stages of the research data life cycle. ^13^ Capacity building has also been an important activity since the establishment of the Marine Metagenomics Community, and many hands-on workshops and training courses have been developed and completed to build competence and expertise in a broader marine academic community.

However, the popularity of metagenomics has continued to grow, with current approaches providing greater genome-resolved insights into the community composition and the functions performed by the microbial constituents, with annotations spanning viruses, bacteria, archaea and microbial eukaryotes. ^14^^–^^18^ Furthermore, metagenomic-like approaches are increasingly being applied to untangle complex holobiont genomes such as lichens, where both the primary symbionts and secondary non-obligate microbes are captured. ^19^ Finally, multi-omics datasets are now being more routinely produced to understand not only the genetic potential, but also the actively produced transcripts, proteins and/or metabolites, with a view to establishing the links between genotype and phenotype. When a host organism is involved, such datasets can also be augmented with genetic data from the host, such as genome, single nucleotide polymorphisms and transcriptomic data. The collective data facilitate a hologenomic approach ^20^ to understanding host phenotypes, in the context of their environment and microbiome. Given this increasing complexity of study designs, and the broad applicability of microbiome research, we advocate expanding the Marine Metagenomics Community to include other areas of microbiome research. In particular, we highlight the need for an ELIXIR Microbiome Community to develop and promote standards and research infrastructures that enable the sharing of efforts, concepts, and best practices, while benefiting from the synergistic interplay with other ELIXIR Communities.

### 2.1 The scope of the ELIXIR Microbiome Community

The term metagenomics is often colloquially applied to many different areas of microbiome research (see Table 1), regularly (incorrectly) used to encompass both shotgun metagenomics (indiscriminate sequencing of DNA from an environmental sample) and metabarcoding approaches (the sequencing of a specific amplified marker gene), as exemplified by the thousands of mislabeled datasets in International Nucleotide Sequence Database Collaboration (INSDC). Depending on the nature of the scientific question being addressed and/or the environment, metagenomic analysis may also involve assembly, and potentially the generation of metagenome assembled genomes (MAGs). ^21^ Equally applicable is the analysis of unassembled raw-read data sets that can be used for taxonomic classification (e.g. Kraken, ^22^ MetaPhlan, ^23^ mOTUs ^24^) and functional profiling approaches that are especially effective when extensive reference databases are available. Sequencing technologies such as long-read sequencing methodologies and the associated adaptive sequencing techniques, ^25^ together with changing protocols such as host material depletion protocols (e.g. ^26^), are facilitating the analysis of a wide-range of differing communities. However, the applicability of certain downstream processing and/or analysis tools changes fundamentally in these different contexts. Similarly with metagenomic data, metatranscriptomic data can be processed in different ways, and with an associated metagenomic dataset from the same sample, enables the estimation of both the genetic potential and actively transcribed fraction. Additionally, metaproteomics and metabolomics are technologies that are increasingly being used in microbiome research, involving the study of proteins expressed or small molecules produced by microbial communities in a given environment, which require quite different methodologies for their analysis.

Fundamentally, the ELIXIR Microbiome Community is about providing the necessary infrastructures required to perform analysis of nucleotide sequence data derived from a microbiome, especially the reproducibility of the results, the archiving and discovery of analyses and the interoperability of tools and data. The Microbiome Community will work with other ELIXIR communities to determine how microbiome-derived data coming from different ‘omics approaches, may be processed and integrated.

**Table 1.** Overview of the terms and techniques used to study microbiome samples.

Term	Definition
Metabarcoding	Amplification and sequencing of diagnostic marker gene(s) found in a microbial community
Metagenomics	Random sequencing of the total DNA found in a microbial community
Metatranscriptomics	As with metagenomics, but the sequencing of the total RNA
Metabolomics (non-targeted)	Indiscriminate study of small molecules and products of metabolism
Metaproteomics	Identification and quantification of proteins and their interactions found in a microbial community

A fundamental challenge for the Microbiome Community is to address the provision of infrastructures that are sufficiently adaptable to permit the most appropriate informatics analysis, depending on the environment sampled and the experiments conducted. Finally, the ELIXIR Microbiome Community will gather a variety of researchers wishing to undertake microbiome research, spanning clinicians aiming to understand the role of the human microbiome in disease aetiology, ecologists wanting to understand the changing landscape of biodiversity, the agritechnology sector wishing to enhance animal and crop production, to biotechnology scientists looking for novel enzymes, among others.

### 2.2 The context within ELIXIR

**Figure 1.**
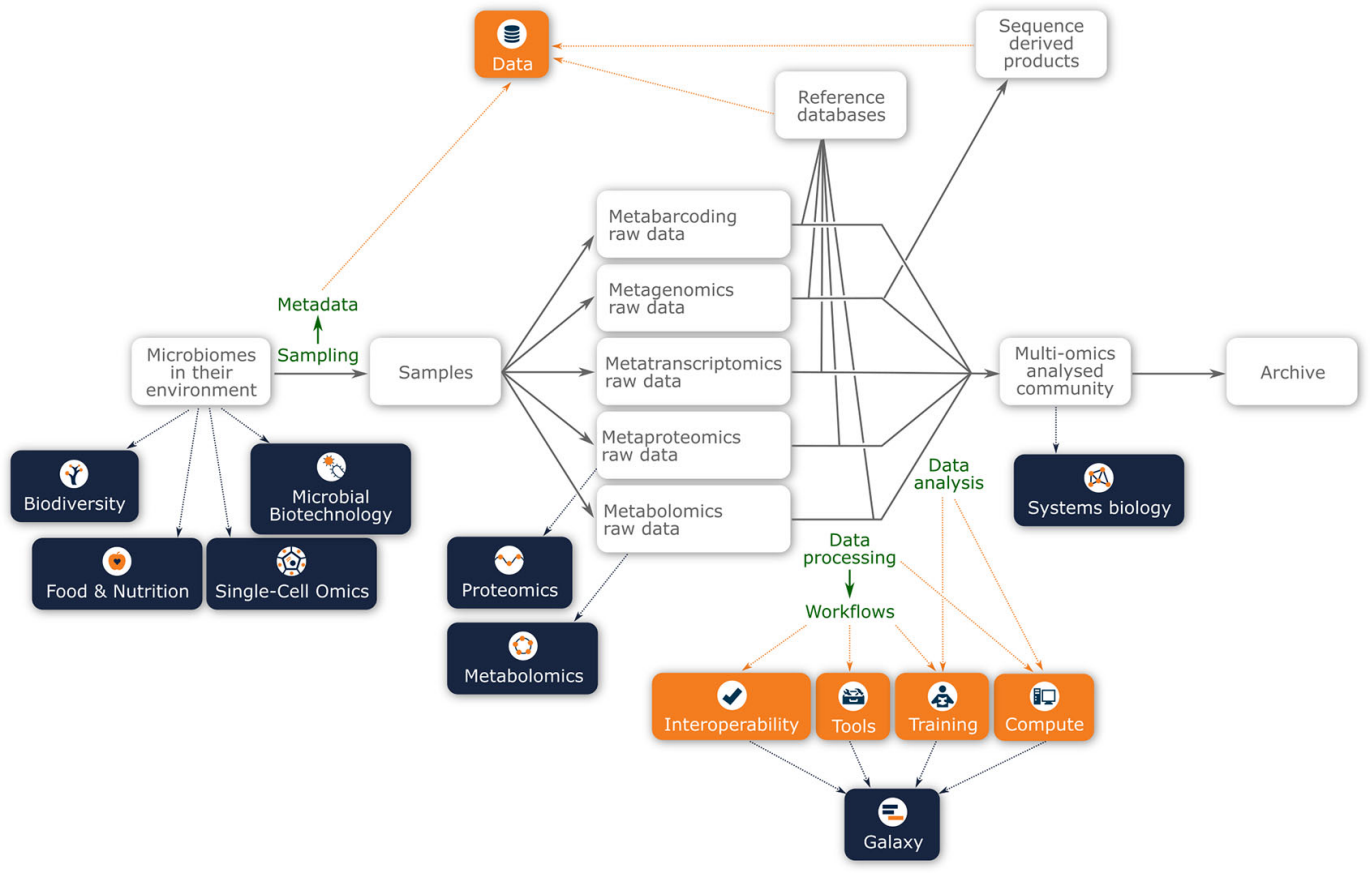
A schematic of how a microbiome sample (i.e. community in the environment) may be analysed using different ‘omics approaches, with the main steps indicated in green. Underpinning these analyses will be the metagenomic and metatranscriptomic data, which will be used as a framework for the metaproteomic and metabolomic interpretation. Highlights in this figure are connections with the ELIXIR platforms (orange boxes) and other ELIXIR communities (dark blue boxes).

Given the breadth of the aforementioned applications of microbiome research, it is unsurprising that there are many links to other current and future ELIXIR activities. Figure 1 presents a schematic layout of the experimental design of a multi-omic analysis of a microbiome sample. Even in this very high-level representation, it can be easily observed that the new ELIXIR Microbiome Community has many potential interactions with other ELIXIR Communities and Platforms along the experimental workflow. Thus, the Microbiome Community represents a showcase of the essence of ELIXIR by bringing together diverse informatics infrastructures that can be coupled together (interoperate) to achieve complex data analyses (on compute infrastructures) that have the appropriate provenance, with data adequately archived in the relevant ELIXIR core data resources. At all levels in ELIXIR, it will be essential to coordinate activities to ensure functional harmony between ELIXIR Communities using Platform-devised solutions.

***2.2.1 Interactions with other ELIXIR Communities***

Some of the key areas of interactions, both ongoing and foreseen, with other Communities are listed in Table 2. As indicated in Figure 1, the interaction with other ELIXIR Communities, specifically those concerned with environmental sampling, begins at the start of the data lifecycle, concerning the sample acquisition and characterisation of the microbial communities. For example, the Food and Nutrition Community aims to understand the relationship between food choices and human health. While microbiome analysis forms part of this Community’s activities, the aim of the Food and Nutrition Community is to integrate microbiome data within the context of food and nutrition data, host genotype and phenotype information, and develop interventions that may impact disease. ^27^ Thus, in the case of the Food and Nutrition Community the microbiome is only a small part of the overall research program, and restricted to human microbiome research. Members of the existing ELIXIR Microbiome Community are already engaged with the Food and Nutrition Community, and have helped to provide microbiome sequence analysis services. Similarly the Biodiversity Community has multiple overlapping activities, but with a distinct remit. For example, computational infrastructures and tools borne out of metagenomics research are now being applied for pathogen and biodiversity surveillance. Furthermore, taxonomic inventories resulting from analysis of metagenomic/metabarcoding data are commonly accepted as biodiversity resources and biodiversity resources such as GBIF and OBIS ^32^ routinely incorporate data from both MGnify and INSDC. Similarly, many of the biodiversity approaches use barcoding methods for studying environmental DNA (eDNA). While this can overlap with the metabarcoding approaches used in the Microbiome Community, eDNA analysis extends to marker genes such as Cytochrome c oxidase subunit I (Cox1) that is specific to macro-organisms and, thus, out of scope for the Microbiome Community and falls into the realm of the ELIXIR Biodiversity Community.

**Table 2.** Overview of existing and planned interactions with ELIXIR Communities.

ELIXIR Community	Existing and planned interaction(s)
3D BioInfo	Improve the organisation, quality control and presentations of protein models (e.g. ESMAtlas ^28^) for proteins predicted from metagenomic and metatranscriptomic assemblies. Improve functional annotations through structure-function-sequence relationships.
Biodiversity	Connect biodiversity/observation resources with ‘omics data/analysis. Identify overlap between analysis pipelines (e.g. barcodes, genome annotations) and promote best practices.
Federated human data	Evaluate the landscape concerning human microbiome and national legislation concerning data sharing. If appropriate, investigate solutions from the Federated human data Community for sharing sensitive data.
Food & Nutrition	Share metagenomics workflows to improve our understanding of the role of the gut microbiome in unlocking nutrients in food.
Galaxy	In collaboration with the Galaxy Community, continue to tailor and expand tools, workflows and training materials applicable to the Microbiome Community, directed by the needs identified by an ongoing evaluation study.
Metabolomics	Develop methods and tools to connect microbiome sequence data (metagenomics and metatranscriptomics) to link functions to metabolites.
Microbial Biotechnology	Improve the identification of valuable enzymatic activities from environmental metagenomics data to identify bioactives (e.g. enzyme, small molecule) of interest for: bioeconomy; applications in food preservation; agriculture; chemistry; or medicine.
Plant Science	Develop a greater understanding of the needs of the Plant Science Community for microbiome-based solutions to improve plant resilience to pathogens, as well as understand how plants maintain their microbial communities across generations, and if so, potential mechanisms for doing so.
Proteomics	Enable the production of tailored reference databases (e.g. biome and/or other contextual metadata) for interpretation of (meta-)proteomics MS2 data. Develop methods to enhance the integration of metagenomic, metatranscriptomics and metaproteomics results.
Single Cell Omics	Explore areas of overlap in data standards ^29^ and annotation pipelines concerning single amplified genomes (SAGs). Increase knowledge within the Microbiome Community concerning spatial single cell data with respect to improving the quality of MAGs/SAGs ^30^ and the identification of microbes in tissues and tumours. ^31^
Systems Biology	Empower a better integration of multi-omics datasets to describe and understand how different community members interoperate to achieve processes. More specifically, ensure that different multi-omics data types from the same sample are appropriately connected across different archive databases, and improve methods for linking metabolomics data to sequence (protein and nucleotide).

With the growing number of multi-omics datasets, establishing strong ties with the ELIXIR Metabolomics and Proteomics communities ^33^ will be essential for understanding how metagenomic and metatranscriptomic data may be utilised by these Communities (e.g. the production of reference databases for the interpretation of the metaproteomics), and the nature of the data types produced by these other ‘omics technologies, their limitations and how the data could be integrated. For example, overlaying metabolomics results on metagenomic data is currently non-trivial due to the scarcity of small molecule annotations that can be linked to functional annotations. Ongoing work with the Microbial Biotechnology and Systems Biology Communities has identified the need to augment the functional annotation of metagenomic and metatranscriptomic data with chemical reaction information from resources such as Rhea. ^34^ While this will improve the discovery of new industrial applications, there is still the need to expand the protein functional annotations of the, so-called, microbial dark matter. The advent of new structural modelling software ^28^^,^^35^ and data resources ^36^ means that there are now structural models for millions of proteins that currently lack functional annotations, yet appear structurally related to functionally characterised proteins. Connections to the 3D BioInfo Community will aid how we store and organise this structural model information, reuse software components for visualisation and leverage their training materials on how to interpret structural model data. This will allow the Microbiome Community to assess the merits and limitations of this data type.

Furthermore, microbiome research has many translational aspects, ranging from the discovery of biomarkers associated with health (of organisms or environments) and disease, to industrial applications such as using enzymes from microbes or the microbes themselves for performing bioremediation and/or replacing chemical processes. One topic that is an area of intensive research is the discovery of enzymes capable of degrading plastics, typically polyethylene terephthalate (commonly known as PET). ^37^ While metagenomic assembly and analysis is providing a rich source of potential new enzymes, the informatics at the core of the Microbiome Community will not provide the information why one enzyme should be assayed in preference to another, how these alpha-beta hydrolases have adapted to utilising PET, or why one enzyme performs better than another. Such answers will come from the collaborative efforts that bridge across Communities, such as microbial biotechnology and 3D BioInfo and, of course, the wider research community.

In summary, there are many synergies and connections between the Microbiome Community and the other existing ELIXIR Communities, but none of these Communities are focused on the core issues concerning microbiome-derived sequence analysis, infrastructure provision, data standards and best practices. Moreover, there are key global societal challenges within the One Health concept defined by the World Health Organization, such as food safety, pathogen tracking, climate changes, antimicrobial resistance (and new therapeutics) and pandemic preparedness, in which microbiome research plays a critical role. Yet each one of these areas is too complex to be tackled individually and therefore requires the collective outputs from more than one ELIXIR Community, and reach far beyond informatics research ( Table 3).

**Table 3.** Description of a selection of current European national and pan-European efforts aimed at microbiome research, and their relevance to the ELIXIR Microbiome Community.

Initiative	Reach	Aims and relevance
M​utualised​ ​Digital​ Spaces​ ​For​ Life​ ​Sciences (MuDIS4LS)	National (FR)	To develop a framework that will connect national and regional data centres to enable the control of biological data from their origin (data-producing national infrastructures) to their public release, while ensuring data security during the intermediate phases of analysis and exploitation. *Relevance*: Guide data management best practices, especially when dealing with sensitive microbiome related data.
Secured computing spaces for the data access and analysis project of the France 2030 programme « Food Systems, Microbiome and Health »(Cloud4SAMS)	National (FR)	To deploy a distributed digital infrastructure enabling researchers to exploit microbiome and health data in a secure computing environment, collating software tools and workflows for processing these data, computing and storage platforms suitable for processing microbiome data and matching them with health data, while respecting data access rules. *Relevance*: This project will define deployment recipes describing all the procedures to instantiate a virtual machine in a secure cloud, install software and transfer datasets. Knowledge developed will guide the deploying of similar distributed infrastructures within ELIXIR.
National Research Center in Bioinformatics for Omics Sciences (CNRBiOmics)	National (IT)	To enhance the ELIXIR Italian node infrastructure, through the establishment of a “centre of excellence” for multi-omics data production, management, and analysis. In addition, establish a higher education training platform to develop skills required to use the infrastructure. *Relevance*: Microbiome multi-omics data produced by the infrastructure will provide example use cases, as well as being a test bed for multi-omics data integration solutions developed by the community.
Consolidation of the Italian Infrastructure for Omics Data and Bioinformatics (ELIXIRxNextGenIT)	National (IT)	To consolidate the ELIXIR-IT infrastructure for omics and bioinformatics, focused on data production, computational analysis, facilities improvement and training, with a view to strengthening the national ELIXIR infrastructure. *Relevance*: Overlaps with training and sharing of microbiome related pipelines.
NFDI4Microbiota	National (DE)	To: (i) promote FAIR principles in the microbiological community; (ii) provide a comprehensive training program; (iii) enhance data resources for microbiology community; (iv) support high-quality research data management; (v) increase data value by standardising and systematically collecting rich metadata and building tools for querying; (vi) make research more reproducible by standardising data processing and analysis; (vii) provide computational tools and infrastructure for the translation of data into new knowledge. *Relevance*: This national programme shares many of the objectives of the Microbiome Community, so it will be important to synergise activities.
European Reference Genomes Atlas (ERGA)	European	To generate eukaryotic reference genomes of European species and create a powerful resource for the understanding of biodiversity. *Relevance*: This collection will also include microbial eukaryotes that can be used to enhance eukaryote genome analysis in microbiome research.
European e-Science Infrastructure for biodiversity and ecosystem research (LifeWatch ERIC)	European	To accelerate the sharing, integration and analysis of open-data and its Virtual Research Environments (VREs) to enable studies on biodiversity structure and conservation related to multiple drivers. *Relevance*: Metagenomics data will be used to develop ecological models, while reference genome data will improve analysis pipelines.
Metaproteomics Initiative	European/ International	To promote dissemination of metaproteomics fundamentals, advancements, and applications through collaborative networking in microbiome research. *Relevance:* The central information hub and open meeting place will allow members of the Microbiome Community to interact with metaproteomics experts. Will help the Microbiome Community aim to standardise and methodologies in this field.
microGalaxy	European/ International	To: (i) develop and sustain microbial data analysis in Galaxy, (ii) implement standardised “best practices”, (iii) expand documentation and training, (iv) coordinate efforts in tools, workflows and training development. *Relevance*: Usable and standardised workflows for the Microbiome Community.

***2.2.2 Interaction with ELIXIR Platforms***

Similar to the collaborations with the ELIXIR Communities, there are multiple ongoing and future interactions with the ELIXIR Platforms. In the following sections the connections between the past Marine Metagenomics Community or the future Microbiome Community and each of the Platforms will be highlighted.

*2.2.2.1 Data*

The aim of the ELIXIR Data Platform is to promote the use, re-use and value of life science data. A key part of this activity has been the establishment of the Core Data Resources (CDR). Underpinning sequenced-based microbiome research is the INSDC, especially the European Nucleotide Archive (ENA) in the context of ELIXIR. Alongside the archived sequence data, users can access comprehensive metadata that is important to contextualise where the data originated. Throughout the lifetime of the ELIXIR Marine Metagenomics Community there have been extensive efforts to increase the standardisation of derived sequence products from metagenomic short-read datasets, particularly increasing the availability of assemblies ^5^ and the introduction of the deposition layers to support the increase in the numbers of MAGs being generated. ^38^ In the new Microbiome Community we will continue to promote and develop these layers to accommodate Eukaryotic MAGs (see below), viral sequences and complex coassembly, as well as incorporating the latest community standards as they are approved by authoritative bodies. The work undertaken to generate the MAR databases highlighted that many marine samples in ENA lack key metadata fields. Through extensive curation efforts, using literature as well as contacting the original data submitters, much of this missing data was retrieved and added to the MAR database. While ENA (or any of the INSDC partners) can not add this metadata to the original sequence record, an ELIXIR sponsored initiative led to the establishment of the Contextual Data Clearinghouse ( CDCH). The CDCH facilitates the capture of additional metadata using controlled vocabularies including a description of how this data was generated (e.g. manual assertion, computationally derived), so that they can be associated with an INSDC record. Longer term, this data will be incorporated into BioSamples.

In other non-sequenced based ‘omics fields, microbiome data archiving and analysis is supported by data-type specific resources. In the case of metaproteomics, the PRIDE database repository (also an ELIXIR CDR) enables archiving and re-analysis of (meta) proteomics data, and now also encourages researchers to upload their metadata in SDRF-format. ^39^^,^^40^ PRIDE is the leading resource of the International ProteomeXchange Consortium of proteomics data resources, involving additional databases in the USA, Japan and China, in addition to PRIDE. Similarly, in the case of metabolites the data can be deposited in the MetaboLights repository ^41^ or similar resources. A current challenge facing the field is connecting different multi-omics data that have been derived from the same sample.

The Data Platform also promotes the linkage between Europe PMC ^42^ and other CDR databases. This is critical for the Microbiome Community as additional contextual metadata can often be found in the literature, ^43^^,^^44^ providing crucial overarching context to the experiment, which can be important for re-analyses or meta-analyses. We will continue to promote such approaches, enriching metadata wherever possible.

Last but not least, new activities will be promoted aimed at the integration of microbiome data coming from different ‘omics approaches. In this context, recently, the PRIDE and MGnify teams developed and implemented new pipelines in both platforms for the re-analysis and integration of metagenomic and metaproteomic data, allowing the re-analysis of metaproteomics datasets from PRIDE using sequence databases generated from MGnify, and contextualising the results back into the MGnify web interface in terms of assembly annotations ( https://github.com/PRIDE-reanalysis/MetaPUF). The ELIXIR Microbiome Community will also work to move the Marine Metagenomics domain in the ELIXIR Research Data Management Kit (RDMKit) towards a more general Microbiome domain.

*2.2.2.2 Tools*

Microbiome data analysis employs a large number of tools which are used to perform basic quality control on the sequence data, with separate tools (and reference databases) typically used for taxonomic and functional profiling. Installing and managing dependencies has been eased by the use of package management systems such as Conda, or through the use of containers, e.g. Singularity. The ELIXIR Microbiome Community will increase their use of BioContainers ^45^ to promote the packaging, containerisation and deployment of tools relevant to microbiome research.

In order to make tools findable by the end users, the Microbiome Community will work on improving their annotation by (i) expanding the EDAM ontology ^46^ to include microbiome-specific keywords, (ii) performing periodic reviews of tools and their associated annotations in the bio.tools ^47^ catalogue. These annotations will subsequently be used to build a catalogue of tools for microbiome data analysis and their availability for different platforms, e.g. Galaxy, ^48^ or as workflow descriptions (e.g. Snakemake, ^49^ CWL, ^9^ Nextflow ^50^), which can be readily combined to make new annotation workflows. Additionally, the Microbiome Community will develop and maintain cloud-deployable and FAIR analysis pipelines using state of the art tools and following best open science practices by: (i) using workflow descriptions; (ii) documenting the workflows and depositing them in WorkflowHub ^51^ for easy discovery, re-use and assessment; (iii) making them available for the Microbiome Community via platforms such as MGnify and Galaxy.

As an integral part of the Tools platform, Galaxy has integration with OpenEBench, WorkflowHub, EDAM, bio.tools and follows all Software Best Practices. A current joint effort between the Microbiome and Galaxy Communities is running an evaluation of tool requirements for microbiome data analysis in the Galaxy ecosystem. This evaluation will lead to a shared roadmap between both Communities for tool integration and standardised workflow development for microbiome data analysis.

A key part of understanding the applicability of a tool/workflow is its benchmarking, and has been an aim of the Tools platform. Very few analyses in microbiome research employ a single tool, with the norm being the coupling of multiple tools and reference databases to achieve a comprehensive analysis that includes both taxonomic and functional results. Even relatively simple workflows that perform metagenomics assembly are computationally heavy. This combination of workflow complexity and typical computational overheads has always made the routine benchmarking tools for microbiome informatics research burdensome. Nevertheless, where two or more tools perform equivalent tasks, it can be relatively simple to modify existing formally described workflows to evaluate their respective performances, but that ease often depends on where they occur in the overall workflow and the metrics used to evaluate the tool. Many efforts have tried to compare the outputs of tools and workflows (e.g. ^47^^,^^51^^–^^54^), with the Critical Assessment of Microbiome Interpretations (CAMI) having become an internationally recognised benchmarking effort. ^55^^–^^58^ The CAMI challenges have established a range of benchmarking datasets for evaluating different categories of tools. Importantly, the organisers of CAMI have engaged data generators to provide data, such that truly independent benchmarking can be undertaken. However, these benchmark datasets can become outdated over time, as the underlying data enters the reference database. The Galaxy Community has already investigated implementing benchmarking infrastructure using CAMI datasets, and increasing the awareness of this infrastructure will be a key effort across Communities and Platforms. As the Microbiome Community establishes, we will develop a broader understanding of the requirements of the wider microbiome research community needs, and feed these requirements to the Tools Platform, as well as seek opportunities to interact with the Tools Platform to capture the diversity of tools and their utility via such benchmarking activities.

*2.2.2.3 Compute*

Depending on the analysis being performed, the computational requirements can be very different. For example, metagenomic assembly typically requires small numbers of cores on a large memory machine, whereas some forms of raw-read analysis require many cores (hundreds) with a small memory footprint. As such, microbiome researchers need to understand the likely computational costs, and their options for deploying them on high performance computing (HPC) and cloud environments. Efforts such as Blue Cloud have helped reduce some of the barriers to using the European Open Science Cloud (EOSC) for marine research through the delivery of a collaborative virtual environment, but the range of services is limited. While such efforts help, there are still many barriers to accessing compute resources and deploying complex metagenomic pipelines in a distributed or even hybrid fashion. Working with the Compute Platform, the ELIXIR Microbiome Community will continue to investigate solutions that facilitate the execution of workflows within such distributed and/or hybrid environments, e.g. using Pulsar network, the distributed compute network offered by the Galaxy Community, and provide guidance of the likely costs of using compute infrastructures.

*2.2.2.4 Interoperability*

Previous work by the Marine Metagenomics Community has leveraged many of the ELIXIR Interoperability Platform solutions, especially the use of workflow languages for the formal description of pipelines, improving the provenance of the data outputs. As such, both the MetaPIPE and MGnify pipelines have been described using the Common Workflow Language (CWL). This effort was paralleled by MG-RAST, ^59^ which also allowed MGnify and MG-RAST to exchange pipelines and establish that the biological signatures reported by the respective pipelines were very similar, yet confounded by different reference databases and methodologies for assigning function. ^60^ Since then, MGnify has published their workflows in WorkflowHub, further promoting their discovery and reuse. As an example of reuse, the MGnify pipeline has been used as the basis for the newly developed metaGOflow pipeline, ^61^ to be used by the Marine Genomic Observatories. Moreover, this work also employed Research Object Crate ( RO-crate ) ^62^ to package relevant metadata about the sample and the bioinformatics analysis applied and the data products. RO-crate offers new opportunities for sharing or federating the metagenomics analysis workload. In parallel, Galaxy, which supports the Tool Registry Service (TRS) protocol to exchange and run workflows between the WorkflowHub and Galaxy, gained support for RO-Crate (version 23.0) to export complete data analysis as a structured and FAIR digital object, supporting the Global Alliance for Genomics and Health (GA4GH) standards, and is in the process of applying to be an ELIXIR Recommended Interoperability Resource.

The Microbiome Community will continue to work with the Interoperability Platform to make wider use of RO-crate, with a view to federate data analysis between resources. For example, future work by the new Microbiome Community will enable the MGnify workflows to be made deployable on Galaxy, with the RO-crate to be transferred, verified and ingested into MGnify. Additional work needs to be undertaken to understand how universal this approach is, so that MGnify could become a hub for a range of additional analyses, thereby reducing the duplication of effort that currently exists in the community.

Finally, we will work on the development of novel mechanisms to integrate and link data coming from multi-omic approaches using different tools and data resources. This will require the development of new data Interoperability layers for data resources that are not normally used in metagenomics-centred Microbiome data, such as the PRIDE database in the case of metaproteomics data.

*2.2.2.5 Training*

One of the key areas commonly highlighted by national and international reports on the potential of microbiome research is the need for training, especially in the area of bioinformatics. As already highlighted, microbiome analysis is an emerging and evolving research field by itself, with plenty of challenges still to be addressed. Combined with this complexity, the increasing number of researchers using such methods makes the need for continuous training and re-training a challenge on its own. Researchers need to become familiar with modern computing technologies, such as HPC and cloud computing, and follow the constant updates on experimental approaches, algorithms (new and updates) and pipeline developments. As new pipelines are established and existing pipelines improved through the incorporation of new tools and/or reference databases, this adds further complexity to the tool and data output landscape associated with microbiome research, and sets the need for well defined and consistent training modules dedicated to computational approaches to microbiome analysis.

Platforms such as MGnify support large-scale services for most steps of a microbiome study, meaning the distribution of raw-data, production of assemblies, their analysis, and their potential use for meta-analysis, have proved of great benefit. Nevertheless, these analyses should be considered just the starting point for further downstream analysis, which requires the specific domain expertise of the researchers involved in undertaking the study. One approach can be the use of cloud-based initiatives such as Galaxy supporting graphical interfaces and allowing the users to choose more specific tools, while tuning their parameters and reference databases according to their environment being studied. Such infrastructures attempt to fill the gap between researchers without experience in computer science and their needs for FAIR and quality microbiome analysis. Despite both solutions being readily available, there remains knowledge gaps and/or reticence about using such resources, often due to a lack of training.

To upskill microbiome scientists and keep them up-to-date in microbiome data analysis and standards, the ELIXIR Microbiome Community will work in coordination with the ELIXIR Training Platform to offer scalable and FAIR training. The Microbiome Community will continue to: (i) annotate training materials with appropriate metadata to create a comprehensive training portfolio; (ii) FAIRify the training content, making it open-access; (iii) register training material, national and international providers and events in ELIXIR’s Training Portal TeSS ^29^; (iv) assist the Training platform in the development of annual training gap surveys; and (v) develop materials and design learning paths specific to different community needs (e.g. biomes or data types).

To enable access to training resources and deliver this training, face-to-face and online workshops will be organised and videos will be recorded for “on demand” learning. The technical infrastructure for training, in particular the computational environment setup and software installation challenge will be addressed in coordination with the ELIXIR Tools and Compute Platforms, with the aim of promoting the use of Conda environments, containers, digital notebooks or platforms like Galaxy which mitigate many of the current obstacles. In order to make these aspirations possible, the Community will increase its training capacity by working with training communities on practices, organising Train the Trainers events and building a community of microbiome research trainers, with areas of expertise covering different environments, ‘omics approaches and data analysis strategies. Ensuring these trainers maintain their knowledge with the evolving informatics landscape is, arguably, a key challenge that is yet to be addressed and something this Community will strive to solve in collaboration with the ELIXIR Training Platform.

## 3. Context with other international initiatives

We have highlighted the need for promoting best practices and standards throughout this article. However, it is also important that the Microbiome Community continues to build upon engagement with organisations such as the Genomics Standards Consortium (GSC ^63^). The GSC has become critical for establishing many of the standards that underpin genomic research, and more recently metabolomic. Examples of GSC established standards that are particularly pertinent to the microbiome domain include: minimal information about any sequence (MIxS ^64^), the Biological Observation Matrix (BIOM) format ^65^; and the Minimum Information about a Metagenome-Assembled Genome (MIMAG ^29^).

There are also other ELIXIR Node-specific initiatives that the Microbiome Community connects with to ensure that the respective efforts are synergised. Examples of projects with ELIXIR Node involvement directly related to the ELIXIR Microbiome Community are presented in Table 3, which cover a diverse range of topics. The engagement needs to be bi-directional to ensure that the needs of Nodes are well understood and that solutions developed at national levels can be spread across the ELIXIR Microbiome Community, and vice versa. In this context, the ELIXIR Microbiome Community leads will undertake coordinating roles, engaging with the project representatives, inviting them to relevant ELIXIR events and promoting active participation in relevant ELIXIR Communities.

MicrobiomeSupport, formerly a European Commission funded coordination and support action (CSA) program aimed at improving microbiome research and innovation, highlighted in their final report ^66^ that there was “limited connectedness” in microbiome research conducted on different environments/systems, and that during the course of this program the lack of connectedness did not improve. This independent finding reinforces the need for broadening the ELIXIR Marine Metagenomics Community to a more generalist Microbiome Community. Since the end of the CSA project, this MicrobiomeSupport has transitioned to the MicrobiomeSupport Association to continue the engagement activities within the microbiome research community and will provide a key dissemination route for the ELIXIR Microbiome Community outcomes.

It will also be important to showcase the ELIXIR Microbiome Community to European countries that are yet to join ELIXIR. For example, Romania has a thriving microbiome research community, but is faced with the same set of informatics challenges. Sharing knowledge beyond ELIXIR, will not be the primary goal, but will nevertheless be important to harmonise the activities internationally and promote the benefits of participation in ELIXIR. Beyond Europe, there are parallel organisations that strive to achieve similar goals to ELIXIR in other locations. For example, Australia BioCommons aims to promote bioinformatics and bioscience data infrastructures at a national level. Given the strength of microbiome research in Australia (see below), we will explore opportunities for international collaboration.

In addition, it will be important to showcase the ELIXIR Microbiome Community to communities (within and outside Europe) that are not yet familiarised with ELIXIR activities. For example, the Metaproteomics Initiative is an international community that promotes dissemination of metaproteomics fundamentals, advancements, and applications through collaborative networking in microbiome research. ^67^^,^^68^ For example, recently, they benchmarked metaproteomics workflows and bioinformatics methods in the field in the first multi-lab benchmark study in metaproteomics (called CAMPI), showcasing the robustness of metaproteomics data analysis workflows. ^67^

Finally, the National Microbiome Data Collaborative (NMDC), ^69^ a US led initiative, is developing a unified data portal to support microbiome multi-omics data integration and analysis through an integrated, distributed framework. Many of the governing principles associated with this portal are common with those described here, especially with the desire to have containerised, reusable computational workflows, as well as trying to make the data compliant with the FAIR principles. Sharing experiences and best practices between NMDC and the ELIXIR Microbiome Community (and others) will improve the global standardisation of microbiome research.

## 4. Interaction with other key data resources beyond ELIXIR

Microbiome research is global, so it is also key that European microbiome research infrastructures are coordinated with other international resources. Below we highlight a small selection of widely used resources that are produced outside Europe, and place them in context of the ELIXIR Microbiome Community. Some of the most utilised tools and resources used by the current Microbiome Community are CheckM, ^70^ the Genome Taxonomy Database (GTDB) and the associated GTDB toolkit. ^71^^,^^72^ CheckM is widely used to assess the completeness and contamination of prokaryotic MAGs, and is part of the GSC reporting standard. The GTDB resources is a genome based taxonomy of prokaryotes, and the associated GTDB-tk facilitates the classification of other prokaryotic genomes against this framework, more often than not, to determine novelty. These are currently made available via the Australian research groups, who face similar challenges in maintaining resources. Other key resources are based in the US, with MG-RAST ^59^ and a range of different resources produced by the Joint Genome Institute (JGI). MG-RAST facilitates the analysis of raw-reads and assemblies (metabarcoding, metagenomics and metatranscriptomics), but does not perform assembly nor offer any form or long-term archiving assurance. The JGI IMG/M resource ^73^ has many parallels with MGnify, offering a wide range of data analyses focused on assembly and MAG generation, but IMG/M does not deal with metabarcoding. Notably, JGI also produces IMG/VR, ^74^ a globally unique collection of viruses, many of which have been determined from metagenomic and metatranscriptomics. Any future effort in Europe focused on viruses must aim to minimise the duplication of effort and content with IMG/VR. Recently it has also produced IMG/PR: a database of plasmids from genomes and metagenomes with rich annotations and metadata. ^75^ Coordinating with these global initiatives is key to ensure the future availability of the tools and resources, ensuring interoperability between the resources, maintaining uniform standards and sharing of the informatics/computational burden.

## 5. Specific challenges and objectives of the ELIXIR Microbiome Community

A key early challenge in developing the ELIXIR Microbiome Community is to establish a detailed understanding of the current approaches and databases used for the analysis of different microbiomes. For example, it is widely accepted that current short-read assembly-based methods do not generally work as well for soil microbiomes due to the diversity of the microbial community typically present (the sequence depths are insufficient to build useful contigs or the datasets are so large, that they are computationally intractable). This current limitation has led and will continue to lead to the development of new experimental methods, from sampling to nucleic acid sequencing and informatics analysis. In this section, some of the key challenges associated with microbiome research are highlighted below, together with how these challenges will be addressed by the new ELIXIR Microbiome Community. Table 4 lists the key thematic areas and objectives that the Microbiome Community will address, split into short-term and longer-term objectives to provide a high-level overview of the proposed activities.

**Table 4.** Objectives of the ELIXIR Microbiome Community.

Area	Objective
**Near-term ** (2 years timeframe)	
Community Expansion	Survey the needs, key datasets, data analysis approaches, ‘omics data types and biome specific specialisation
	Identify key experts involved in viral, prokaryotic and eukaryotic analysis
	Establish and share a strategic technical roadmap with the Communities and Platforms, highlighting key contacts
	Identify relevant funding calls, with the aid of building microbiome research informatics capacities and connecting to key experts in other ‘omics (e.g. metaproteomics)
Training	Increase awareness of microbiome tools, resources, and their applicability to different microbiomes
	Address knowledge gaps in generating and adopting data analysis workflows
	Teach advanced containerisation and cloud deployment
Co-ordinate	Increase rates of data archival deposition, with rich contextual metadata. Establish a mapping between biome and checklists
	Promote data analysis through the use of ELIXIR services
	Share of ideas on the design and implementation of workflows for microbiome research, promoting the use of best practices
	Organise in-person and virtual meetings for the Microbiome Community
Industry connection	Use ELIXIR and Node forums to understand pharmaceutical and biotechnological demands and current limitations impacting this sector.
**Longer-term ** (~3-5 years)	
Training	Design targeted training for different microbiome communities
	Addressing the issue of maintaining “Train the Trainer”
	Organise hackathon to improve integration of ELIXIR services providing microbiome data
	Establish a rich set of training materials, appropriately tagged to aid find ability
Federated data analysis	Enable the execution of MGnify pipelines in Galaxy and/or other data management workflows, and submission of results to MGnify
	Establish routine mechanisms for federating microbiome analysis (e.g. RO-Crates, resources)
	Demonstrate approaches to multi’omics integration, through collaborative, cross-Community initiatives
Promoting new approaches	Establish new standards for microbiome research, particularly with respect to data analysis reporting and contextual metadata reporting in conjunction with GSC
	Leverage new data-types and experimental approaches to improve the scope and/or quality of microbiome analysis
	Enhance existing or establish new reference databases in response to the Microbiome Community demand and capacity
	Establish new methods for across study comparisons, mitigating against confounding factors to enhance discovery
	Provide a mechanism for estimating the cost/benefit of performing different types of analysis in the context of different microbiomes
International harmonisation	Represent the Microbiome Community at international conferences, promoting the Community/ELIXIR outputs and solutions
	Foster international collaborations between other resources providers and databases to ensure global harmonisation of e-infrastructures for microbiome research
	Leverage the CAMI initiative to facilitate benchmarking of tools and workflows

The Microbiome Community will also provide a mechanism for sharing knowledge about new approaches for microbiome research, be it experimental or informatics-based techniques. For example, there is an increasing number of metagenomics datasets that are produced using long-read sequence technologies. While long-read sequencing technologies can require larger quantities of DNA or may be more error prone compared to third-generation short-read sequencing technologies – which can limit their use – the long-reads can mitigate the computational burden of metagenomic assembly and increase the confidence in analysis results (e.g. MAGs produced by long-reads can have high contiguity and therefore less prone to contamination). The long-reads can be paired with short-read sequences, which can then be used in different ways (e.g. sequence error correction). Increasing the awareness of these long-read and hybrid-sequencing approaches, the workflows that support their analysis and when and where they could be applied will be a key output of the Microbiome Community. Similarly, there are other experimental approaches such as single amplified genomes (SAGs), which have increased in popularity. The Microbiome Community will also be important for assessing the utility of emerging sequencing approaches, such as adaptive sequencing approaches. In this case, the methods can access low abundance microbes, although such methods will not facilitate the generation of abundance profiles. Bringing these data types alongside the ubiquitous short-read datasets will require new standards and data integration approaches to be developed by the Microbiome Community.

There has been a paradigm-shift in metagenomic analysis with a common goal now being the generation of environmental genomes (MAGs), which has not only allowed the identification of thousands of specific functions, but facilitated them to be assigned to specific organisms. As such, this has started the development of specific MAG deposition layers, ^76^ and the development of MAG specific resources. The new Microbiome Community will promote the use of MAG deposition, and provide guidelines and software to aid their deposition. Workflows that encompass both MAG generation and quality verification will be developed that include the capture of both prokaryotic and eukaryotic MAGs. The Microbiome Community will help establish best practices for eukaryotic MAG discovery, as well as develop new standards for removing redundancy and methods for assigning taxonomy, which are recognised gaps in the area of eukaryotic MAG discovery. While prokaryotic MAG recovery methods are more mature and standardised, it is anticipated that there will be continuous improvements in both experimental and computational methods for generating longer contigs, and more datasets that enable different approaches to enhance the detection of contamination and/or misassembly. The ELIXIR Microbiome Community will also evaluate methods and establish best practices for the identification of sub-species/strains in metagenomic datasets. To do so, we will engage with efforts such as the CAMI ^55^^,^^77^ to identify tools that can scalably and accurately classify MAGs at a finer grain taxonomic level than species.

Finally, the classification and naming of MAGs is going to be paramount, so that the novel biodiversity can be understood and more easily referenced by the scientific community. Currently, the Microbiome Community has widely adopted the GTDB ^71^ and the associated GTDB-tk ^72^ for classifying MAGs against a reference tree. However, the taxonomy of GTDB differs from the more widely-used NCBI taxonomy, and there is a need to increase the interoperability between these two taxonomies. The ELIXIR Microbiome Community will work on addressing the current issues associated with MAGs and taxonomy. Additionally, another key area of development of taxonomy will be increasing the linkage between genomic resources and marker genes, such as the ribosomal small subunit (SSU) RNA.

In addition to cellular microbes, another area for the ELIXIR Microbiome Community to address is the development of the infrastructure and resources for identifying and cataloguing viruses in metagenomic and metatranscriptomic data. ^78^^–^^81^ Viral genomes are incredibly diverse in terms of composition and organisation. However, there are three challenges associated with viral microbiomes: (i) there is no universal marker gene covering all viruses; (ii) viral taxonomic frameworks are incomplete; (iii) there is no centralised database collecting the millions of viral sequences; and (iv) metagenomics informatics often only produces fragments of viruses, which causes ambiguities concerning their classification and functions. It will be critical for the ELIXIR Microbiome Community to engage with established viral infrastructures and organisations, such as the European Virus Bioinformatics Center, to establish methods, standards and resources for improving the analysis of viruses found in microbiome sequence data, and how best to overcome the current fragmented organisation of viral datasets.

The increase in metagenomic assemblies has resulted in a parallel increase in the number of predicted protein sequences that have been identified, with sets of non-redundant proteins now in the billions. There is huge potential for discovery in these protein datasets, as well as de novo designs fit for purpose, e.g. carbonic anhydrases ^82^ and a key aim for the new ELIXIR Microbiome Community will be ensuring that these data are annotated, both as individual sequences or as higher order grouping (e.g. pathways, biosynthetic gene clusters). This will involve the evaluation of emerging tools, as well as harnessing structural models to allow the detection of relationships that are undetectable by current sequence based methods. The Microbiome Community will need to work together to shed light on the functions of the so-called ‘Dark Matter’, develop standards for functional labelling that encapsulate both the mechanisms and confidence of the annotation, and develop new infrastructural frameworks for accessing slices of the data based or adequate representatives based on the requirements.

As identified by the ELIXIR Marine Metagenomics Community, experimental and contextual metadata is critical to comparative metagenomics. The absence of rich contextual and experimental metadata limits data reuse and the production of downstream data products, such as assemblies and MAGs. With the Microbiome Community, we will identify areas where metadata standards need to be improved, with biome specific contextual metadata being the most likely source of specific metadata checklist. The Microbiome Community will develop training promoting the need for metadata, checking compliance against standards, how the metadata can be captured and submitted to accompany the sequence data, and potentially other ‘omics data types. Within the Microbiome Community, we will promote and develop standards regarding the analysis provenance, and how the collective corpus of metadata can be used to improve meta-analysis and the identification of confounding factors when comparing different research projects.

Another key challenge that the Microbiome Community needs to address is ensuring that compute resources are accessible for performing data analysis that can be associated with microbiome derived sequence data. Previously, we have highlighted the need for interaction with the ELIXIR Compute, Interoperability and Training platforms, as well as ELIXIR Communities such as the Galaxy Community. This requires that analysis pipelines are readily discoverable and deployable, and that key issues regarding both compute processing and storage requirements are well understood. Additionally, given that microbiome associated data analysis has such computational overheads, it is vital that models for data archiving and/or sharing are developed by the Microbiome Community to increase the capacity of microbiome research within Europe. This may require the extensions to existing databases or development of new ones, but it requires an agreement from the research community to adopt them. Achieving this will involve both communication and training of the microbiome research community.

While there are data resources such as MGnify that provide access to consistent analyses pertaining to different metabarcoding, metatranscriptomics and metagenomics datasets from a variety of biomes, it is fundamental to remember that these data outputs do not represent the end of the analysis pathway. Typically studies require comparison between different cohort groups (disease vs health, treatment vs non-treatment). Furthermore, as the biological signal from meta’omics datasets can be extremely noisy, there can often be the need to combine datasets to boost statistical significance of the biological signal. Similarly, the combination of studies can also be used to: (i) contextualise against previous studies (e.g. similar studies on the same diseases); (ii) understand the distribution of microbes or functional features (e.g. antimicrobial resistance genes) between different geographical locations; and/or (iii) study the relationship between biomes (e.g. studies adopting a One Health approach). To enable such large, complex studies there needs to be a greater understanding of the approaches suitable for cross study comparisons, and their limitations. Thus, a major objective for the Microbiome Community will be to include those researchers that are developing methods that can identify and mitigate experimental and informatic confounding factors, which currently limit data reuse. Existing approaches often rely on correlating contextual and experimental metadata with statistically significant factors identified in the datasets. There is also the need to develop and promote methods for performing robust statistical analysis of microbiome derived data, thereby enabling biological signals to be extracted from cross-sample/project datasets. Currently, there is a tendency to analyse the different ‘omics datasets independently, and then correlate the derived signals. However, statistical methods are being developed to facilitate the analysis of integrated multi-omics datasets, and it will be important that the Microbiome Community determines the applicability of these approaches for microbiome research.

In the context of other ‘omics approaches, there are also some major challenges in metaproteomics. ^83^ One of the major challenges is the construction of tailored protein sequence databases which are needed to identify proteins in complex microbial communities. Metaproteomics aims to elucidate the functional and taxonomic interplay of proteins in microbiomes, but the diversity and vast number of unknown and uncharacterized proteins present in these communities makes database creation and accurate protein identification difficult. As microbial communities are highly dynamic and their protein expression can vary significantly, conventional protein sequence databases might not cover the entire diversity, leading to potential limitations in accurate protein identification (e.g. the use of de novo sequencing). Other challenges in metaproteomics includes how to report proteins which cannot be explained by a corresponding metagenomic study, so called unidentified proteins of unknown function, methods for protein inference and quantification that are comparable across studies, the dynamic range of protein sizes analysed, as well as how researchers handle non-specific peptide-spectrum matches, compared to specific matches. Addressing these challenges is crucial for improving the reliability and confidence of metaproteomic analysis and obtaining comprehensive insights into the functional roles of proteins in complex microbiomes.

As metagenomic methods have become a more routine method for studying microbial communities, metagenomics has been and will continue to be paired with more and more diverse sets of measurements of the microbiome. Examples of non-omics data collected alongside metagenomics data include geochemical (e.g. PANGAEA ^84^) measurements, meteorological, image data and even acoustics. While methods are already emerging for the integration of ‘omics datatypes (e.g. MOFA, ^85^ MIA ( https://github.com/microbiome/mia)), integration of these additional non-omics data types will enable a broader understanding of microbiomes in context. For the new Microbiome Community, it will be essential to identify the appropriate archives for these data types, and establish the methods to facilitate navigating between datasets from the same samples. Only through achieving this, can new data visualisation schemas that enable the combination of environmental, geospatial and temporal data, in addition to biological data (taxonomy/function), be developed.

## 6. Conclusions

The overarching aim of the Microbiome Community is to develop a sustainable bioinformatics infrastructure for microbiome resources (data, tools, workflows, standards, training) which will enable a deep understanding of the function and taxonomy of the entire microbial fraction. The aim is to be biome-agnostic and, balanced in supporting the analysis and interpretation of data from different environments. We aim to highlight the very best approaches for the analysis and integration of different data types (e.g. sequences, metabolites, proteomics, and images) and their visualisation. By broadening the Marine Metagenomics Community we will engage many more researchers and aspire to have a greater representation of scientists from different disciplines, such as ecologists and clinicians, complementing the strong molecular biology and genomics backgrounds already represented in the Microbiome Community. The Microbiome Community will have key roles in engaging with policy makers (e.g. access and benefit sharing, climate change impact assessment), as well as the industrial sector, which is increasing the translation of basic research to microbiome-based products (e.g. UK Microbiome Strategic Roadmap for Innovation). Such a strong microbiome infrastructure as envisaged by the Microbiome Community is essential to maximise the impact that European research programs have in the field of microbiome research, and to facilitate the exploitation of microbiome-based solutions in a range of settings, from clinical to industrial processes, thereby addressing key societal challenges and needs.

## Data Availability

No data is associated with this article *.*
