# Supplementary material for: Establishing the ELIXIR Microbiome Community
Source: F1000Res. 2025 Sep 8;13:ELIXIR-50. Originally published 2024 Jan 8. [Version 2] doi: 10.12688/f1000research.144515.2 (PMC12441670; doi:10.12688/f1000research.144515.2)
Supplement: Peer review history

# Reviewer report: Reviewer response for version 2

**DOI:** 10.5256/f1000research.185171.r412934

**Published:** 2025-10-06

**Authors:** Lauren Lui

**Affiliations:** 1 E O Lawrence Berkeley National Laboratory, Berkeley, California, USA

Summary

*Finn et al*. have written a comprehensive white paper on the establishment of the ELIXIR Microbiome Community.  They outline the purpose of establishing the ELIXIR Microbiome Community, the history of its formation, interaction with other ELIXIR communities, plans for data management and computational resources, and challenges in implementation.  I found the manuscript informative about the intent of the ELIXIR Microbiome Community initiatives, and it was useful to have the historical and international context included. There is a lot of information packed into this manuscript!  I only have minor issues with the manuscript that I think can easily be addressed.

Major Comments

 On page 6, the authors state that the ELIXIR Microbiome Community is about providing the necessary infrastructure for “nucleotide sequence data” from a microbiome.  In the context of the entire manuscript this statement is confusing because proteomics and metabolomics are also discussed in relation to the entire ELIXIR metacommunity.  Should this statement be revised or can there be some clarification added here?

Why I put “no” for “Are all factual statements correct and adequately supported by citations?” and why I put “no” for “Are arguments sufficiently supported by evidence from the published literature”

 There are some blanket statements in the manuscript that are consistent with the literature but there aren’t any citations. There are a few statements that are incorrect.

- Introduction.  There are many statements here that could be supported by literature references.  For example, the statement that viruses that infect bacteria play critical roles in community dynamics could easily have a few references.  This statement could also be expanded to include archaeal and eukaryotic viruses.  For example, viruses that infect eukaryotic algae are also very important in the dynamics of algal blooms. The statement about the impact of dysbiosis of microbiomes could also have a few references.
- One Health.  One Health is used in the manuscript but not defined nor does it have a citation.  Please describe briefly and include a citation. Page 8.
- Microbial dark matter. There’s at least two places that mentions “microbial dark matter” but don’t define it (pages 8, and 17).  Capitalization is not consistent between these two instances.  Please include a reference and/or define this term.
- Page 16.  Most of the content on this page could use more references:
  - Section on long reads.
    - This secion could include citations on long read metagenomics studies, notably the publication of Microflora Danica from  Mads Albertsen’s lab (https://www.nature.com/articles/s41564-025-02062-z).  I acknowledge that this came out fairly recently and the authors may have drafted this section before this publication came out, but there are many other long read metagenome papers that can also be cited.
    - My second issue with this section is that long reads don’t really mitigate the computational burden – you still can’t assemble the metagenomes on a laptop and nanopore basecalling needs GPUs.  Long reads assist with genome assembly because the reads are longer than genomic repeats, eliminating the ambiguities of short read assembly. This is discussed by Ryan Wick on his github page (https://github.com/rrwick/Unicycler?tab=readme-ov-file) and in the introduction and conclusion of this paper https://pmc.ncbi.nlm.nih.gov/articles/PMC8172020/.
  - Paragraph on MAGs.
    - MAGs should be defined as “metagenome-assembled genomes” not “environmental genomes.” MAGs can also be from clinical metagenomes. 
    - In the same sentence as above, the statement “has allowed the identification of thousands of specific functions” is unclear as to what it refers to.  If this refers to protein gene function, then all of these would be by computational prediction and not really identification of new functions.  A citation would be helpful here.
  - Paragraph about classification of MAGs. 
    - Please add “taxonomic” in front of “classification” in the first sentence for clarity
    - There’s been huge changes in prokaryotic taxonomy in recent years, and there are no citations to the committee reports here or opinion pieces of the use of “candidatus” when naming organisms.  It is worth a few sentences of discussion here or added references.
  - Paragraph on viruses
    - Please add a few references for the statements about the challenges of analyzing viral microbiomes.
    - The statement that there is no centralized database is incorrect.  IMG/VR, which is mentioned in the article, has millions of viral genomes.

Some statements may need to be qualified or rephrased.

- Page 11, the statement “Even relatively simple workflows that perform metagenomics assembly are computationally heavy.” This statement is heavily qualified based on someone’s experience with and access to computational resources, as well as the amount of data to be processed.  It would also be better to use something like “resource-intensive” instead of “heavy.”  If this statement could also be changed to something like “most metagenomes cannot be assembled on a laptop” to provide more context for the reader.
- Page 17, last paragraph.  The sentence about the challenges in metaproteomics mentions proteins that are not identified in corresponding metagenomes and calls them “so called unidentified proteins of unknown function.” This seems to be a bit of a non sequitur because proteins of unknown function are those that cannot have a function assigned computationally, not because they are not found in the genomic data. It is also possible that "so called unidentified proteins of unknown function" is a separate list item - if so then numbers should be added into this sentence for clarity.
- Page 14, the statement beginning with “For example, it is widely accepted that current short-read assembly-based methods…” The accuracy of this statement depends on access to computational resources.  Terabase scale metagenomes have been done ( https://www.nature.com/articles/s41598-020-67416-5).  Perhaps change “computationally intractable” to “highly computationally resource intensive.”

Minor comments

*Use of “ELIXIR Microbiome Community”*

- In most places, “ELIXIR Microbiome Community”  is used, but in some places it is only “Microbiome Community.” Please change all of these instances to “ELIXIR Microbiome Community” for consistency.

*ELIXIR Platforms*

- Please add a table of what each of the platforms do or describe briefly in the text (top of page 5)

*ASV*

- ASV is the abbreviation for “amplicon sequence variant” not “amplified sequence variant”

*Typos*

- Page 11, “non-sequenced” should be “non-sequence”’
- Page 11, bottom of paragraph 4, should microbiome be lower case?

*Citations*

- INSDC needs a citation (bottom of page 5)

*Figure 1*

- I assume that the starting point for this diagram is the “Microbiomes in their environment” box.  This could either be a unique color (so something other than white, blue, or orange) or larger than the other boxes to visually indicate that this is the starting point of the data/material workflow.

*Abbreviations*

 Given the nature of this article, there are many acronyms and the definitions of a few were missed. 

- GBIF – page 8
- Country abbreviations in Table 3 (FR, IT, DE)

*Grammar*

 There are some grammatical issues sprinkled throughout the manuscript but are easily fixed:

1. Colons and semicolons are misused throughout this manuscript given the large number of lists.  Colons only come after complete clauses.  Semi-colons can only separate two independent clauses. For example, on page 4, the last sentence of the first paragraph should be something like “Key challenges facing the microbiome research community are how to (i) appropriately store the data, (ii) informatically process, integrate, compare and interpret microbiome-derived data, and (iii) how to make the data findable, accessible, interoperable, and reusable, *i.e., *FAIR.”  The colon was removed and commas used to separate the list items.  This is also an issue in the third paragraph of section 2.2.2.5 Training, first paragraph of section 3, page 16 fifth paragraph, fourth paragraph on page 17, last row of table 3.
2. Use commas after i.e. and e.g.
3. I think that last sentence of the third paragraph on page 6 may need to have an “and” in front of “to biotechnology”.
4. Page 17, last paragraph.  The sentence about the challenges in metaproteomics could use numbers for the items. As it is currently structured, the sentence is confusing to read.
5. Page 18.  Second paragraph would benefit from the use of the oxford comma.  Example is add a comma after “image data.” The last sentence of this paragraph is awkwardly worded.

Is the topic of the opinion article discussed accurately in the context of the current literature?

Yes

Are arguments sufficiently supported by evidence from the published literature?

No

Are all factual statements correct and adequately supported by citations?

No

Are the conclusions drawn balanced and justified on the basis of the presented arguments?

Yes

Reviewer Expertise:

Microbial ecology, Bioinformatics, Metagenomics

I confirm that I have read this submission and believe that I have an appropriate level of expertise to confirm that it is of an acceptable scientific standard, however I have significant reservations, as outlined above.

# Reviewer report: Reviewer response for version 2

**DOI:** 10.5256/f1000research.185171.r412360

**Published:** 2025-09-16

**Authors:** Charlie Pauvert

**Affiliations:** 1 University Hospital of RWTH, Aachen, Germany

Thanks to the authors for addressing all my comments and suggestions. They successfully restructured and amended the tables 2 and 3 and the article for an increased impact to highlight how the scientific community can benefit from the ELIXIR Microbiome Community.

 On a small minor note, it seems possible to acknowledge the authors of the MIA package more than the URL by citing their work see https://microbiome.github.io/mia/authors.html#citation (and the added citation #1 via the form).

Is the topic of the opinion article discussed accurately in the context of the current literature?

Yes

Are arguments sufficiently supported by evidence from the published literature?

Partly

Are all factual statements correct and adequately supported by citations?

Partly

Are the conclusions drawn balanced and justified on the basis of the presented arguments?

Partly

Reviewer Expertise:

microbiome, bioinformatics, reproducible research, data and metadata standards

I confirm that I have read this submission and believe that I have an appropriate level of expertise to confirm that it is of an acceptable scientific standard.

## References

[1] : mia: Microbiome analysis. R package version 1.17.9. *https://microbiome.github.io/mia/* .2025;

# Reviewer report: Reviewer response for version 1

**DOI:** 10.5256/f1000research.158321.r251099

**Published:** 2024-05-11

**Authors:** Almut Heinken

**Affiliations:** 1 University of Lorraine, Lorraine, France

In this article, the ELIXIR Microbiome community is described. The community was established in 2015 as the Marine Metagenomics community and was recently expanded to the scope of metagenomics research for multiple areas such as human health, agriculture, and ecology. The authors then describe their perspective for the community. One focus area will be the promotion of best practices for metagenomics analyses by benchmarking tools. Interoperability between different tools will also be facilitated. Another focus will be encouraging data sharing and reuse and providing data storage platforms. Finally, links to existing ELIXIR initiatives in related areas as well as with international initiatives will be established.

 Overall, this is a very clear, concise, and informative review. The scope and aims of ELIXIR Microbiome are well-described and detailed. The short-term and long-term objectives are also clearly described.

 Specific comments:

- I appreciate the links to other ELIXIR initiatives in Table 2. I would be particularly interested in more detail on the integration with the Systems Biology community. How would the ability to reuse multi-omics data for systems biology approaches be improved?
- It is a bit unclear to me which is the proposed data platforms, tools, and training will be freely available to non-ELIXIR members.
- Regarding providing metadata of samples, how will GDPR regulations be handled for human samples?

Is the topic of the opinion article discussed accurately in the context of the current literature?

Yes

Are arguments sufficiently supported by evidence from the published literature?

Yes

Are all factual statements correct and adequately supported by citations?

Yes

Are the conclusions drawn balanced and justified on the basis of the presented arguments?

Yes

Reviewer Expertise:

Systems biology

I confirm that I have read this submission and believe that I have an appropriate level of expertise to confirm that it is of an acceptable scientific standard.

# Response

**Published:** 2025-07-15

**Authors:** Bérénice Batut

**Affiliations:** Institut Francais de Bioinformatique, Évry-Courcouronnes, Île-de-France, France

*In this article, the ELIXIR Microbiome community is described. The community was established in 2015 as the Marine Metagenomics community and was recently expanded to the scope of metagenomics research for multiple areas such as human health, agriculture, and ecology. The authors then describe their perspective for the community. One focus area will be the promotion of best practices for metagenomics analyses by benchmarking tools. Interoperability between different tools will also be facilitated. Another focus will be encouraging data sharing and reuse and providing data storage platforms. Finally, links to existing ELIXIR initiatives in related areas as well as with international initiatives will be established.*

*Overall, this is a very clear, concise, and informative review. The scope and aims of ELIXIR Microbiome are well-described and detailed. The short-term and long-term objectives are also clearly described.*

**We would like to thank the reviewer for their positive comments concerning the ELIXIR microbiome community papers. Below we address their specific comments.**

*Specific comments:*

- *I appreciate the links to other ELIXIR initiatives in Table 2. I would be particularly interested in more detail on the integration with the Systems Biology community. How would the ability to reuse multi-omics data for systems biology approaches be improved?*

**We have added a few sentences to expand how the integration might be improved.**

- *It is a bit unclear to me which is the proposed data platforms, tools, and training will be freely available to non-ELIXIR members.*

**All of the proposed activities are freely available to non-ELIXIR members. ELIXIR does not put explicit boundaries on who can use, but engagement with some ELIXIR events may be preferentially given to scientists coming from ELIXIR member states and funds from ELIXIR funding schemes would be restricted to member states.**

- *Regarding providing metadata of samples, how will GDPR regulations be handled for human samples?*

**The landscape concerning human microbiome samples is complicated. Currently there is little consensus across Europe whether human microbiomes should be under controlled access. Similarly, the GDPR landscape is also complicated and not entirely independent.  There are already established routes for suppression of data (should an individual wish to be forgotten), which can be propagated to other databases (e.g. MGnify will remove analyses associated with a suppressed sequence dataset). This is clearly an area that will need to be developed as part of the ELIXR community, as no specific solutions have been agreed, we would prefer not to comment about how they will be handled in this manuscript.**

# Reviewer report: Reviewer response for version 1

**DOI:** 10.5256/f1000research.158321.r244855

**Published:** 2024-03-14

**Authors:** Charlie Pauvert

**Affiliations:** 1 University Hospital of RWTH, Aachen, Germany

The authors make the case for mutating the ELIXIR Marine Metagenomics Community initiative into an ELIXIR Microbiome Community. It is indeed timely to have an such a pan-European initiative especially as it draws on previous experience, albeit a more narrow biome. The emphasis on multi-omics integration is also a strong suit as it is a complex topic where the microbiome research community would need support and infrastructure. The authors map out existing (but not all) similar initiatives, even if it is unclear how they would work together. In my opinion, a couple of claims should be strengthened and the argumentative power of this white paper would benefit from minor reorganization, a text slim down and extra proofreading. To this end, I highlight the strengths and weaknesses of the manuscript below.

 ## Strengths

 - Table 3 is a great idea to map out the others initiatives but needs a bit rework to be straight to the point.

 - In the Data section, the part between "Throughout the lifetime of the ELIXIR Marine Metagenomics Community" and the end of the paragraph is really relevant and draws from the experience of the ELIXIR Community. These sentences should be more highlighted, maybe upstream. I do wonder how the authors considered how the others initiatives (mentioned in Table 3) could also contribute to the addition of metadata for a global curation effort, possibly via the mentioned Contextual Data Clearinghouse.

 - The data re-analysis and integration between PRIDE and MGnify (named MetaPUF) is a good use case of the efforts that the new ELIXIR Community can promote.

 - I did appreciate the clear objective at the end of the Compute section, to help with scaling up analyses as well as the plan for the interaction with the ELIXIR Training Platform which promise to give a boost in training the current and next generation of microbiome researchers.

 - I liked how the authors highlighted (using the example of viral sequences databases) that time and resources should not be wasted in duplicating works, and that initiatives such as the ELIXIR Microbiome Community can promote this.

 - The authors thought ahead to promote and develop methods to limit confounding factors when re-using datasets, especially in multi-omics settings, and added this to one of the ELIXIR Microbiome Community challenges.

 ## Weaknesses

 ### Introduction

 - (minor) The microbiome definition used in the paper was revisited in Review reference 1 to include the interactions as well as others molecules than genomes as part of the microbiome. I suggest to use this definition given the emphasis on additional omics made in the paper.

 - (major) The first paragraph ends on challenges of our research community including how to make data FAIR. Given the experience gained by the ELIXIR Marine Metagenomics Community, I would have appreciated a sentence on why making our data FAIR is important (e.g., transparency to make the research more reproducible, accountability given the (public) source of funding). See major comment in next section.

 ### The scope of the ELIXIR Microbiome Community

 - (major) These following arguments for FAIR data should be in the Introduction section.

 "Moreover, when wishing to contextualise the results with similar experiments, the way a dataset has been produced and processed must be transparent to establish whether it is comparable (e.g. amplified sequence variants can only be compared when the same amplified regions are compared). Furthermore, when different methods are applied, best practices in data stewardship are required to ensure that the connectivity of the derived sequence data products, together with functional and taxonomic assertions are kept in context of the original sample/sequencing effort and associated contextual metadata."

 - (minor) The very first sentence of this section blames researchers for misuse of a term "[...] regularly (incorrectly) used [...]". I suggest to rephrase the sentence to simply state the differences, or back up the misuse of the term with references or a survey.

 - (minor) "Fundamentally, the ELIXIR Microbiome Community is about providing the necessary infrastructures required to perform analysis of **nucleotide** sequence data derived from a microbiome"

 - (minor) "Finally, and possible unique to this ELIXIR Community, is the variety of researchers". I am not sure that these features are unique to the ELIXIR Community but rather to the field of microbiome research. I suggest to tone it down by simply pointing out that the ELIXIR Community gathers a variety of researchers.

 ### Table 1

 - (major) Metabolomics is listed in Table 1 but is omitted from the narrative in the paragraph.

 - (major) The Table 1 is not really used but could actually reduce some redundancies in the manuscript by providing a one-stop-shop to explain and detail these techniques.

 ### Figure 1

 - (major) The figure is early on in the manuscript and it is unclear which of the ELIXIR platforms and communities are already established, or foreseen. Especially since "current and future ELIXIR activities" is mentioned in the manuscript before referencing this figure. The Table 2 does not add more information in that regard.

 - (major) There is a need for different types of arrows as the same arrow represent interactions between communities or processes.

 - (minor) The "Data" node is quite generic, I was wondering whether it meant public repositories, institute repositories or both. Please be precise.

 - (minor) I guess the type of metadata illustrated in the figure is restricted to biological metadata, that is indeed collected during sampling, however, technical metadata such as the sequencing method used, the type of instrument or the library layout, are collected during the *processing* of the sample not only the sampling itself.

 - (minor) There should be an arrow from "Archive" back to "Data" when the data produced is deposited and then contributes back to public repositories?

 ### Interactions with other ELIXIR Communities

 * (major) The sentence "Similarly, many of the biodiversity approaches use marker gene amplification for studying environmental DNA (eDNA)" is redundant with the previous one **and** introduces a different nomenclature that was not used before  (marker gene amplication vs metabarcoding) and the differences, if any, are not explained. I would suggest to remove the sentence.

 * (major) The term "Isolation of genomes" is misleading and I guess the authors used it as a shorthand for "The isolation of bacteria, its DNA extraction, genome sequencing and their annotation". Please rephrase to avoid misinterpretation. Plus I would argue that these steps are also done when deconstructing microbiomes via cultivation strategies and are therefore not so out-of-scope.

 * (minor) "yet each one of these areas is far greater in scientific scope" feels exaggerated and vague. It should be rephrased. A suggestion is: "yet each one of these areas is too complex to be tacked individually"

 * (minor) There is no link nor transition between the paragraph that starts with "In summary, " before the Table 3 and the paragraph after that starts with "Similarly, microbiome research". Please rephrase or edit to connect the two sections. Plus, whilst this is good to have a concrete example in the "Similarly" paragraph, the paragraph before was very broad and doing a summary. I would suggest to try reordering the two paragraphs and bring the example earlier for a smoother transition.

 * (minor) Typo "Similarly, microbiome research has many translation **al** aspects"

 * (minor) The PET acronym is detailed but actually used only once, I am unsure if this is necessary.

 ### Table 2

 - (major) The text in the "Interaction" column needs rework as it does not use a consistent wording and could be more to the point, especially as it is a complement to the main text:

     - The Food and Nutrition entry is a question.

     - The Galaxy entry has an unnecessary return carriage, and a unspecific "ongoing evaluation study" that needs to be clarified.

     - The Plant Science entry starts with a generic sentence that could be in the introduction or removed for clarity. Plus I would add a clarification that "plants maintain **or not** their microbial communities across generations."

 - (minor) The status (e.g., currently active, inactive, planned, etc.) of each ELIXIR Communities would have been appreciated as it is missing also from the Figure 1.

 - (minor) The mention of "the field" for the Federated human data community is vague in a manuscript about gathering communities, which research field is implied? If this is the human microbiome research field as a whole, please indicate.

 ### Table 3

 - (major) Similarly to the Table 2 major comment, there is a lack of consistent wording in the "Aim" column that makes the Table 3 not as impactful as it should and could be. Maybe the authors could extract common/distinct features from each of these initiatives as an alternative way to the "Aim" column. A couple of suggestions for these features would be: is it a national initiative?, does it relates to data storage, data analysis, training? is it linked to ELIXIR?

 - (major) I was surprised not to see the NMDC listed in the table 3, especially when it is discussed in the main text. What about the NCCR Microbiomes initiative in Switzerland? I can understand that some initiatives are not included for space reason, but maybe state it in the legend of the table.

 - (minor) Is this table sorted? It seems not, but it could be by acronyms or names.

 - (minor) The aim for the NFDI4Microbiota is way too big a paragraph. The authors should reduce it for conciseness.

 - (minor) The Metaproteomics Initiative entry has a hyperlink and a reference when none of the others have. Please homogenise.

 - (minor) Some entries have country listed and some not. Please homogenise.

 - (minor) I am not questioning the existence of the European Reference Genome Atlas here, but how best to phrase its relevance to ELIXIR in the manuscript. There seems to have no prokaryotes genomes in their atlas, however, there seems to be a trove of fungi and protists genomes which are usually said to be understudied in microbiome. So I think there is a missed opportunity for the authors here to make the most out of this entry.

 - (minor) "With its headquarters in Bari (Apulia region)," seems irrelevant in the context of the table, please remove.

 ### Data

 - (minor) The end of the following sentence is redundant as the INSDC was introduced earlier already "INSDC, which in collaboration with the National Institute of Genetics DNA DataBank of Japan (DDBJ) and the United States National Center for Biotechnology’s (NCBI) GenBank and Sequence Read Archive (SRA), facilitate the deposition and global exchange of sequence data.". Please adjust accordingly.

 - (minor) "A current challenge facing the field is connecting different multi ‘omics data that have been derived from the same sample." Is this going to be tackled by the ELIXIR Microbiome Community? If so, I would state that this is part of its objectives.

 - (minor) The end of sentence "[...]overarching context to the experiment, which can be important for meta-analyses." seems like an euphemism, I would suggest to replace with "[...]overarching context to the experiment, re-analyses or meta-analyses." to include re-analyses as well.

 - (minor) "We will continue to promote such approaches, enriching metadata wherever possible." Is this going to be done via the CDCH?

 - (minor) "The ELIXIR Microbiome Community will also work to move the Marine Metagenomics domain in the RDMKit towards a more general Microbiome domain." What is the RDMKit? It is not explained, nor cited nor mentioned again.

 ### Tools

 - (minor) "will increase their use of BioContainers" should be "will increase the use of BioContainers"

 - (minor) "In order to make tools findable **by the end users, ** the Community"

 - (minor) "workflow descriptions (e.g. Snakemake, CWL, Nextflow)" None of them have their references cited, is it an omission or space limitation?

 - (minor) "A **current** joint effort between the Microbiome and Galaxy Communities"

 ### Benchmarking

 - (major) "Benchmarking" it is the only item at this hierarchy level, meaning that this is useless for structuring the text. Please edit.

 - (minor) Review reference 2 published a recent review with guidelines to learn from that could have its place in this paragraph.

 - (minor) in the sentence: "As the Microbiome Community establishes, we will develop a broader understanding of the requirements of the **Community**, feed this to the Tools Platform, as well as seek opportunities to interact with the Tools Platform to capture the diversity of tools and their utility via such benchmarking activities.", is this the ELIXIR Microbiome Community, or the wide community of microbiome researchers? Is it to mean that the ELIXIR Microbiome Community is going to act as an interface between microbiome researchers and ELIXIR Tools/Infrastructure?

 ### Compute

 - (minor) The first sentence would fit better in the introduction.

 ### Interoperability

 - (minor) The reference 57 should be at the end of the sentence starting with "This effort was paralleled" not in the middle.

 - (minor) Reference 58 should be removed at it is a duplicate of reference 47.

 - (minor) Use the full text "Global Alliance for Genomics and Health" instead of GA4GH.

 - (minor) "is in the process of applying to be a **n ELIXIR** Recommended Interoperability Resource."

 - (minor) In the sentence: "This will require the development of new data Interoperability layers for data resources that are not normally focused in Microbiome data" I think the authors meant "used" instead of "focused", and "microbiome" instead of "Microbiome".

 ### Training

 - (minor) In "Platforms such as MGnify support large-scale services for most, if not all, steps of a microbiome study", I would suggest to remove the "if not all".

 - (minor) "with areas of expertise covering different environments, ‘omics approaches and data analysis pathways." I think the authors meant "learning paths", and I would refrain from using "pathways" as it also has a biological meaning.

 ### Context with other international initiatives

 - (major) Whilst I appreciated the emphasis that no initiative exists on its own, I feel the first paragraph on the Genom **ic** (please correct the typo) Standards Consortium feels lengthy for a manuscript whose topic is not the GSC. I would advise to summarize. In this respect, the second paragraph is particularly relevant to a tangible collaboration between ELIXIR Microbiome Community and GSC.

 - (major) The NMDC is discussed in this section but not part of the Table 3.

 - (minor) Is the mentioned M5 project still active as the website's last update is 2012?

 - (minor) "Combining the activities on standards concerning workflows [...]" does this means adding and providing workflows to the microbiome research community?

 - (minor) Given the emphasis on the fact that MicrobiomeSupport *was* a program, the authors could update the readers and indicate that it is now MicrobiomeSupport Association.

 ### Interaction with other key data resources beyond ELIXIR

 - (major) There is an order issue with the main text that a proofread could solve, as MG-RAST is explained and cited in the first paragraph but already mentioned upstream of the main text in the Interoperability section.

 ### Specific challenges and objectives of the ELIXIR Microbiome Community

 - (major) The strong claim "it is *widely accepted* that current short-read assembly-based methods do not generally work as well for soil microbiomes" would probably need at least one reference.

 - (major) "(iii) there is no centralised database collecting the millions of viral sequences". It seems to be the case indeed, and there are databases (~24) out there as recently compiled in Review reference 3. How ELIXIR Microbiome Community plans to integrate/aggregate these resources in a non-duplicating manner?

 - (minor) The word "through" is superfluous in the the sentence that starts with "This current limitation, [...]" and can be removed.

 - (minor) The CAMI was already explained and cited above, so the already defined acronym can be used.

 - (minor) Precise the area in :"Additionally, another key area of development **of taxonomy** [...]".

 - (minor) The sentence "Viruses, particularly those that infect bacteria, are found ubiquitously in all environments and play critical roles in community dynamics." belongs in an introduction, not so downstream of the manuscript.

 - (minor) The sentence starting the sixth paragraph could be precised as "The increase in metagenomic assemblies has resulted in a parallel increase in the number of **predicted** protein sequences, with sets of non-redundant proteins now in the billions."

 - (minor) Fix typo in "that are undetectable by current sequence based methods."

 - (minor) Would it make sense to also be able to access representative, of clusters for instance? "[...] develop new infrastructural frameworks for accessing slices of the data **or adequate representatives** based on the requirements."

 - (minor) What is the " **expanded** Microbiome Community"? It was never mentioned before.

 - (minor) A few comments on the sentence: "Within the Community, we will develop and promote standards around the analysis provenance (analytical metadata),". In my opinion and how it was already stated in the manuscript, it would make more sense to promote existing standards first and then develop if need be. There is no mention of other type of metadata, so the "analytical metadata" precision seems superfluous. I would suggest: "Within the Community, we will **promote and develop** standards **regarding** the analysis provenance,"

 - (minor) Precise the term forms in "[...] ensuring that comput **ing** resources are accessible for performing the different *forms* of data analysis [...]", do the authors mean types of/steps in the data analysis?

 - (minor) Same argument as before regarding reinventing the wheel, I would swap the part of the sentence: "This may require the **extensions to existing databases or development of new ones**, but it requires an agreement from the research community to adopt them."

 - (minor) This part "Metaproteomics aims to elucidate the functional and taxonomic interplay of proteins in microbiomes," should have been in the Table 1, or to reuse the Table 1 here.

 - (minor) The tenth paragraph of this section starts with the mention of multiple major challenges, but detail "only" one of them. I would suggest to mention some of the others challenges.

 - (minor) The "MIA" method is just a hyperlink, without any reference. Either cite the website accordingly or add the reference.

 ### Table 4

 - (major) I was surprised to see that the objective "Foster international collaborations between other resources providers and databases to ensure global harmonisation of e-infrastructures for microbiome research" was long-term, as I would have imagined that a gap analysis would be short-term to ensure we do not reinvent the wheel, especially given the others initiatives discussed in the manuscript.

 - (minor) If the Objective column starts with action verbs (which is a good idea), then it should be "Survey the needs" instead of "Survey of needs".

 - (minor) Specify the type of workflow with "Address knowledge gaps in generating and adopting **data analysis** workflows"

 - (minor) The verb is missing in " **Teach a**dvanced containerisation and cloud deployment"

 - (minor) The verb is missing in " **Promote** data analysis through the use of services". Is this ELIXIR services in general or specific ELIXIR Microbiome Community services?

 - (minor) Use "Share" instead of "Sharing" in the "Co-ordinate" entry.

 - (minor) The "Industry connection" entry does not fit the action verb pattern. A suggestion would be "Use ELIXIR and Node forums to understand pharmaceutical and biotechnological demands and current limitations impacting this sector." as the first sentence felt generic.

 - (minor) The verb is missing in " **Design** targeted training for different microbiome communities"

 - (minor) Use "findability" instead of "discoverability" for consistency and to fit with the FAIR principles.

 - (minor) Reorder the sentence to start with the action verb: " **E**stablish new standards for microbiome research, particularly with respect to data analysis reporting and contextual metadata reporting **in conjunction with GSC**"

 - (minor) Correct "Established" to "Establish" in the "Promoting new approaches" entry.

 Editorial comments:

 - (major) "Community" is used in upper-case and this is unclear in many instances whether the ELIXIR Marine Metagenomics Community is referred to, the ELIXIR Microbiome Community, or the broader microbiome research community. I suggest to use consistently the full term for the sake of transparency. Abbreviations like EMMC and EMC could be even more misleading in my opinion.

 - (major) The structure of the white paper is not evident as the hierarchy is indicated only by change in font size, and some sections are quite lengthy for a white paper that is supposed to be concise. I understand that this is a constraint from the Editor, but see Review reference 4 for a white paper with a more clearer structure. An alternative could be to use numbered sections.

 - References

     - (minor) The very first reference is oddly formatted in the text creating an artificial and confusing end of sentence.

     - (minor) Title of reference 9 is truncated and should be "Methods included: standardizing computational reuse and portability with the Common Workflow Language"

     - (minor) The superscript numbers of the numeric style of bibliography are in many instances after the final dot (see reference 2, 12-16, 17-19, 36, 37), after a comma (see reference 10, 20, 23), a bracket (see reference 60) or semi colon (see reference 65) when they should be before any of these symbols.

     - (minor) In the paragraph "Interactions with other ELIXIR Communities", we jump from reference 24 to 29 when the numeric style of bibliography (that was chosen by the authors) is expected to mirror the mentions in the manuscript. Please either have the Table 2 earlier in the paper, or change the order of the references.

Is the topic of the opinion article discussed accurately in the context of the current literature?

Yes

Are arguments sufficiently supported by evidence from the published literature?

Partly

Are all factual statements correct and adequately supported by citations?

Partly

Are the conclusions drawn balanced and justified on the basis of the presented arguments?

Partly

Reviewer Expertise:

microbiome, bioinformatics, reproducible research, data and metadata standards

I confirm that I have read this submission and believe that I have an appropriate level of expertise to confirm that it is of an acceptable scientific standard, however I have significant reservations, as outlined above.

## References

[1] : Microbiome definition re-visited: old concepts and new challenges. *Microbiome* .2020;8(1) : 10.1186/s40168-020-00875-0 10.1186/s40168-020-00875-0 32605663 PMC7329523

[2] : Navigating the Landscape: A Comprehensive Review of Current Virus Databases. *Viruses* .2023;15(9) : 10.3390/v15091834 10.3390/v15091834 37766241 PMC10537806

[3] : Challenges and best practices in omics benchmarking. *Nat Rev Genet* .2024; 10.1038/s41576-023-00679-6 10.1038/s41576-023-00679-6 38216661

[4] : A community proposal to integrate proteomics activities in ELIXIR. *F1000Res* .2017;6: 10.12688/f1000research.11751.1 10.12688/f1000research.11751.1 28713550 PMC5499783

# Response

**Published:** 2025-07-15

**Authors:** Bérénice Batut

**Affiliations:** Institut Francais de Bioinformatique, Évry-Courcouronnes, Île-de-France, France

*The authors make the case for mutating the ELIXIR Marine Metagenomics Community initiative into an ELIXIR Microbiome Community. It is indeed timely to have an such a pan-European initiative especially as it draws on previous experience, albeit a more narrow biome. The emphasis on multi-omics integration is also a strong suit as it is a complex topic where the microbiome research community would need support and infrastructure. The authors map out existing (but not all) similar initiatives, even if it is unclear how they would work together. In my opinion, a couple of claims should be strengthened and the argumentative power of this white paper would benefit from minor reorganization, a text slim down and extra proofreading. To this end, I highlight the strengths and weaknesses of the manuscript below.*

*## Strengths*

*- Table 3 is a great idea to map out the others initiatives but needs a bit rework to be straight to the point.*

*- In the Data section, the part between "Throughout the lifetime of the ELIXIR Marine Metagenomics Community" and the end of the paragraph is really relevant and draws from the experience of the ELIXIR Community. These sentences should be more highlighted, maybe upstream. I do wonder how the authors considered how the others initiatives (mentioned in Table 3) could also contribute to the addition of metadata for a global curation effort, possibly via the mentioned Contextual Data Clearinghouse.*

*- The data re-analysis and integration between PRIDE and MGnify (named MetaPUF) is a good use case of the efforts that the new ELIXIR Community can promote.*

*- I did appreciate the clear objective at the end of the Compute section, to help with scaling up analyses as well as the plan for the interaction with the ELIXIR Training Platform which promise to give a boost in training the current and next generation of microbiome researchers.*

*- I liked how the authors highlighted (using the example of viral sequences databases) that time and resources should not be wasted in duplicating works, and that initiatives such as the ELIXIR Microbiome Community can promote this.*

*- The authors thought ahead to promote and develop methods to limit confounding factors when re-using datasets, especially in multi-omics settings, and added this to one of the ELIXIR Microbiome Community challenges.*

*## Weaknesses*

*### Introduction*

*- (minor) The microbiome definition used in the paper was revisited in Review reference 1 to include the interactions as well as others molecules than genomes as part of the microbiome. I suggest to use this definition given the emphasis on additional omics made in the paper.*

**We have added that the definition includes other molecules in addition to genomes.**

*- (major) The first paragraph ends on challenges of our research community including how to make data FAIR. Given the experience gained by the ELIXIR Marine Metagenomics Community, I would have appreciated a sentence on why making our data FAIR is important (e.g., transparency to make the research more reproducible, accountability given the (public) source of funding). See major comment in next section.*

**Thank you for this suggestion. We have added a few sentences to this effect in the manuscript.**

 ### The scope of the ELIXIR Microbiome Community

*- (major) These following arguments for FAIR data should be in the Introduction section.*

*"Moreover, when wishing to contextualise the results with similar experiments, the way a dataset has been produced and processed must be transparent to establish whether it is comparable (e.g. amplified sequence variants can only be compared when the same amplified regions are compared). Furthermore, when different methods are applied, best practices in data stewardship are required to ensure that the connectivity of the derived sequence data products, together with functional and taxonomic assertions are kept in context of the original sample/sequencing effort and associated contextual metadata."*

**We have merged this part of the paragraph into the Introduction.**

*- (minor) The very first sentence of this section blames researchers for misuse of a term "[...] regularly (incorrectly) used [...]". I suggest to rephrase the sentence to simply state the differences, or back up the misuse of the term with references or a survey.*

**We have qualified this with an example of INSDC mislabelling.**

*- (minor) "Fundamentally, the ELIXIR Microbiome Community is about providing the necessary infrastructures required to perform analysis of **nucleotide** sequence data derived from a microbiome"*

**“Nucleotide” has been added to this sentence.**

*- (minor) "Finally, and possible unique to this ELIXIR Community, is the variety of researchers". I am not sure that these features are unique to the ELIXIR Community but rather to the field of microbiome research. I suggest to tone it down by simply pointing out that the ELIXIR Community gathers a variety of researchers.*

**Modified accordingly.**

*### Table 1*

*- (major) Metabolomics is listed in Table 1 but is omitted from the narrative in the paragraph.*

**We have added a sentence in the paragraph.**

*- (major) The Table 1 is not really used but could actually reduce some redundancies in the manuscript by providing a one-stop-shop to explain and detail these techniques.*

**We have increased the cross linking to the table.**

 ### Figure 1

 - (major) The figure is early on in the manuscript and it is unclear which of the ELIXIR platforms and communities are already established, or foreseen. Especially since "current and future ELIXIR activities" is mentioned in the manuscript before referencing this figure. The Table 2 does not add more information in that regard.

*- (major) There is a need for different types of arrows as the same arrow represent interactions between communities or processes.*

**Modified accordingly.**

*- (minor) The "Data" node is quite generic, I was wondering whether it meant public repositories, institute repositories or both. Please be precise.*

**"Data" refers here to the ELIXIR Data Platform**

*- (minor) I guess the type of metadata illustrated in the figure is restricted to biological metadata, that is indeed collected during sampling, however, technical metadata such as the sequencing method used, the type of instrument or the library layout, are collected during the processing of the sample not only the sampling itself.*

**This is everything from phenotypic, sample conditions, experimental methods**

*- (minor) There should be an arrow from "Archive" back to "Data" when the data produced is deposited and then contributes back to public repositories?*

*### Interactions with other ELIXIR Communities*

* (major) The sentence "Similarly, many of the biodiversity approaches use marker gene amplification for studying environmental DNA (eDNA)" is redundant with the previous one **and** introduces a different nomenclature that was not used before  (marker gene amplication vs metabarcoding) and the differences, if any, are not explained. I would suggest to remove the sentence.

**We have modified the sentence to refer to barcoding rather than removing the sentence. We feel it is important to mention eDNA.**

* (major) The term "Isolation of genomes" is misleading and I guess the authors used it as a shorthand for "The isolation of bacteria, its DNA extraction, genome sequencing and their annotation". Please rephrase to avoid misinterpretation. Plus I would argue that these steps are also done when deconstructing microbiomes via cultivation strategies and are therefore not so out-of-scope.

**This sentence has been removed as the reviewer is correct that cultivation of bacteria (and other organisms) from microbiomes is becoming an increasing trend.**

* (minor) "yet each one of these areas is far greater in scientific scope" feels exaggerated and vague. It should be rephrased. A suggestion is: "yet each one of these areas is too complex to be tacked individually"

**We have amended the sentence accordingly.**

* (minor) There is no link nor transition between the paragraph that starts with "In summary, " before the Table 3 and the paragraph after that starts with "Similarly, microbiome research". Please rephrase or edit to connect the two sections. Plus, whilst this is good to have a concrete example in the "Similarly" paragraph, the paragraph before was very broad and doing a summary. I would suggest to try reordering the two paragraphs and bring the example earlier for a smoother transition.

**The paragraphs have been reordered as suggested and slightly amended to improve readability.**

* (minor) Typo "Similarly, microbiome research has many translation **al** aspects"

**Fixed**

* (minor) The PET acronym is detailed but actually used only once, I am unsure if this is necessary.

**There is another instance of this abbreviation, so we have retained this abbreviation.**

*### Table 2*

*- (major) The text in the "Interaction" column needs rework as it does not use a consistent wording and could be more to the point, especially as it is a complement to the main text:*

*- The Food and Nutrition entry is a question.*

*- The Galaxy entry has an unnecessary return carriage, and a unspecific "ongoing evaluation study" that needs to be clarified.*

*- The Plant Science entry starts with a generic sentence that could be in the introduction or removed for clarity. Plus I would add a clarification that "plants maintain **or not** their microbial communities across generations."*

*- (minor) The status (e.g., currently active, inactive, planned, etc.) of each ELIXIR Communities would have been appreciated as it is missing also from the Figure 1.*

*- (minor) The mention of "the field" for the Federated human data community is vague in a manuscript about gathering communities, which research field is implied? If this is the human microbiome research field as a whole, please indicate.*

**In response to all of the above comments, we have substantially reworked table 2. The only comment that we have not addressed so specifically, is whether an activity is in progress or not, because the activities ebb and flow, and it is not always easy to say when there is a start or an end. However, being somewhat more focused in the content, we feel this revised table provides a stronger view of the direction that the community wants to take with the other communities.**

*### Table 3*

*- (major) Similarly to the Table 2 major comment, there is a lack of consistent wording in the "Aim" column that makes the Table 3 not as impactful as it should and could be. Maybe the authors could extract common/distinct features from each of these initiatives as an alternative way to the "Aim" column. A couple of suggestions for these features would be: is it a national initiative?, does it relates to data storage, data analysis, training? is it linked to ELIXIR?*

*- (major) I was surprised not to see the NMDC listed in the table 3, especially when it is discussed in the main text. What about the NCCR Microbiomes initiative in Switzerland? I can understand that some initiatives are not included for space reason, but maybe state it in the legend of the table.*

*- (minor) Is this table sorted? It seems not, but it could be by acronyms or names.*

*- (minor) The aim for the NFDI4Microbiota is way too big a paragraph. The authors should reduce it for conciseness.*

*- (minor) The Metaproteomics Initiative entry has a hyperlink and a reference when none of the others have. Please homogenise.*

*- (minor) Some entries have country listed and some not. Please homogenise.*

*- (minor) I am not questioning the existence of the European Reference Genome Atlas here, but how best to phrase its relevance to ELIXIR in the manuscript. There seems to have no prokaryotes genomes in their atlas, however, there seems to be a trove of fungi and protists genomes which are usually said to be understudied in microbiome. So I think there is a missed opportunity for the authors here to make the most out of this entry.*

*- (minor) "With its headquarters in Bari (Apulia region)," seems irrelevant in the context of the table, please remove.*

**As with Table 2, we have substantially reworked Table 3, including ordering by the reach of the effort, harmonising the language and reducing text. We have also included the relevance of the activity to the community. We have not included NMDC, as this is a US effort, and while important to the community, this table is focused on Europe efforts.**

*### Data*

*- (minor) The end of the following sentence is redundant as the INSDC was introduced earlier already "INSDC, which in collaboration with the National Institute of Genetics DNA DataBank of Japan (DDBJ) and the United States National Center for Biotechnology’s (NCBI) GenBank and Sequence Read Archive (SRA), facilitate the deposition and global exchange of sequence data.". Please adjust accordingly.*

**We have removed the redundancy here.**

*- (minor) "A current challenge facing the field is connecting different multi ‘omics data that have been derived from the same sample." Is this going to be tackled by the ELIXIR Microbiome Community? If so, I would state that this is part of its objectives.*

**We feel this is likely to be a joint effort across different communities. Thus, we have retained the sentence as is.**

*- (minor) The end of sentence "[...]overarching context to the experiment, which can be important for meta-analyses." seems like an euphemism, I would suggest to replace with "[...]overarching context to the experiment, re-analyses or meta-analyses." to include re-analyses as well.*

**We have added “re-analyses or” as suggested.**

*- (minor) "We will continue to promote such approaches, enriching metadata wherever possible." Is this going to be done via the CDCH?*

**While CDCH offers one approach, we believe that there are many different ways that metadata may be enriched, first by making scientists more aware of the need of submitting meta, promoting different, more specific checklists (e.g. STORMS or MicroB3) and mining metadata from published literature. This, specifically highlighting CDCH would not be appropriate.**

 - (minor) "The ELIXIR Microbiome Community will also work to move the Marine Metagenomics domain in the RDMKit towards a more general Microbiome domain." What is the RDMKit? It is not explained, nor cited nor mentioned again.

**This has been expanded to the ELIXIR Research Data Management Kit and a link has been added to the text.**

*### Tools*

*- (minor) "will increase their use of BioContainers" should be "will increase the use of BioContainers"*

**Corrected.**

*- (minor) "In order to make tools findable **by the end users, ** the Community"*

**Done.**

*- (minor) "workflow descriptions (e.g. Snakemake, CWL, Nextflow)" None of them have their references cited, is it an omission or space limitation?*

**Added references**

*- (minor) "A **current** joint effort between the Microbiome and Galaxy Communities"*

**Fixed**

*### Benchmarking*

*- (major) "Benchmarking" it is the only item at this hierarchy level, meaning that this is useless for structuring the text. Please edit.*

**Thank you for pointing this out. We have changed the sub-sub-heading into an introductory sentence to this paragraph.**

*- (minor) Review reference 2 published a recent review with guidelines to learn from that could have its place in this paragraph.*

*- (minor) in the sentence: "As the Microbiome Community establishes, we will develop a broader understanding of the requirements of the **Community**, feed this to the Tools Platform, as well as seek opportunities to interact with the Tools Platform to capture the diversity of tools and their utility via such benchmarking activities.", is this the ELIXIR Microbiome Community, or the wide community of microbiome researchers? Is it to mean that the ELIXIR Microbiome Community is going to act as an interface between microbiome researchers and ELIXIR Tools/Infrastructure?*

**We clarified this to mean the wider community of microbiome researchers.**

*### Compute*

*- (minor) The first sentence would fit better in the introduction.*

*### Interoperability*

*- (minor) The reference 57 should be at the end of the sentence starting with "This effort was paralleled" not in the middle.*

**This citation has been moved to the end of the sentence.**

 - (minor) Reference 58 should be removed at it is a duplicate of reference 47.

**References have been fixed**

*- (minor) Use the full text "Global Alliance for Genomics and Health" instead of GA4GH.*

**This abbreviation has been expanded.**

*- (minor) "is in the process of applying to be a **n ELIXIR** Recommended Interoperability Resource."*

**ELIXIR has been added to this sentence.**

*- (minor) In the sentence: "This will require the development of new data Interoperability layers for data resources that are not normally focused in Microbiome data" I think the authors meant "used" instead of "focused", and "microbiome" instead of "Microbiome".*

**We have updated the sentence accordingly.**

*### Training*

*- (minor) In "Platforms such as MGnify support large-scale services for most, if not all, steps of a microbiome study", I would suggest to remove the "if not all".*

**Agreed.**

*- (minor) "with areas of expertise covering different environments, ‘omics approaches and data analysis pathways." I think the authors meant "learning paths", and I would refrain from using "pathways" as it also has a biological meaning.*

**Actually, we do mean data analysis, but have changed to data analysis strategies.  This is in reference to the fact that there may be multiple different ways of analysing a data type, for example metagenomics can be analysed using tools such as Kraken or MetaPhlAn4, to full assembly, gene calling and functional analysis.**

*### Context with other international initiatives*

*- (major) Whilst I appreciated the emphasis that no initiative exists on its own, I feel the first paragraph on the Genom **ic** (please correct the typo) Standards Consortium feels lengthy for a manuscript whose topic is not the GSC. I would advise to summarize. In this respect, the second paragraph is particularly relevant to a tangible collaboration between ELIXIR Microbiome Community and GSC.*

**The typo has been corrected.**

*- (major) The NMDC is discussed in this section but not part of the Table 3.*

**Table 3 lists pan-European efforts, whereas the NMDC is a US specific initiative. Thus, we have not added this to the table.**

*- (minor) Is the mentioned M5 project still active as the website's last update is 2012?*

**While the website has not been updated, there are ongoing efforts to expand and revitalise this effort. RDF is a member of the GSC board and is promoting aspects of the M5 initiative.  While the name may change, we feel this is useful to keep.  However, as part of condensing the overall length of the manuscript, we have removed the reference to this initiative.**

*- (minor) "Combining the activities on standards concerning workflows [...]" does this means adding and providing workflows to the microbiome research community?*

*- (minor) Given the emphasis on the fact that MicrobiomeSupport was a program, the authors could update the readers and indicate that it is now MicrobiomeSupport Association.*

*### Interaction with other key data resources beyond ELIXIR*

*- (major) There is an order issue with the main text that a proofread could solve, as MG-RAST is explained and cited in the first paragraph but already mentioned upstream of the main text in the Interoperability section.*

**This has now been fixed.**

 ### Specific challenges and objectives of the ELIXIR Microbiome Community

 - (major) The strong claim "it is *widely accepted* that current short-read assembly-based methods do not generally work as well for soil microbiomes" would probably need at least one reference.

**Added**

*- (major) "(iii) there is no centralised database collecting the millions of viral sequences". It seems to be the case indeed, and there are databases (~24) out there as recently compiled in Review reference 3. How*

*ELIXIR Microbiome Community plans to integrate/aggregate these resources in a non-duplicating manner?*

*- (minor) The word "through" is superfluous in the the sentence that starts with "This current limitation, [...]" and can be removed.*

**We have removed the sentence “through”.**

*- (minor) The CAMI was already explained and cited above, so the already defined acronym can be used.*

**This has been fixed.**

*- (minor) Precise the area in :"Additionally, another key area of development **of taxonomy** [...]".*

**Added**

*- (minor) The sentence "Viruses, particularly those that infect bacteria, are found ubiquitously in all environments and play critical roles in community dynamics." belongs in an introduction, not so downstream of the manuscript.*

*- (minor) The sentence starting the sixth paragraph could be precised as "The increase in metagenomic assemblies has resulted in a parallel increase in the number of **predicted** protein sequences, with sets of non-redundant proteins now in the billions."*

**Added “predicted” to the sentence.**

*- (minor) Fix typo in "that are undetectable by current sequence based methods."*

**Fixed - sequenced -> sequence**

*- (minor) Would it make sense to also be able to access representative, of clusters for instance? "[...] develop new infrastructural frameworks for accessing slices of the data **or adequate representatives** based on the requirements."*

**Added** 

*- (minor) What is the " **expanded** Microbiome Community"? It was never mentioned before.*

**Removed “expanded” from the sentence.**

*- (minor) A few comments on the sentence: "Within the Community, we will develop and promote standards around the analysis provenance (analytical metadata),". In my opinion and how it was already stated in the manuscript, it would make more sense to promote existing standards first and then develop if need be. There is no mention of other type of metadata, so the "analytical metadata" precision seems superfluous. I would suggest: "Within the Community, we will **promote and develop** standards **regarding** the analysis provenance,"*

**We agree with the reviewer's comment and have re-ordered accordingly.**

*- (minor) Precise the term forms in "[...] ensuring that comput **ing** resources are accessible for performing the different forms of data analysis [...]", do the authors mean types of/steps in the data analysis?*

**“compute resources” is an accepted resource.  We have removed the “different forms of” as we feel it is a little superfluous.  We were meaning the different strategies, but it does not add to the sentence.**

*- (minor) Same argument as before regarding reinventing the wheel, I would swap the part of the sentence:*

*"This may require the **extensions to existing databases or development of new ones**, but it requires an agreement from the research community to adopt them."*

**We agree, and have amended accordingly.**

*- (minor) This part "Metaproteomics aims to elucidate the functional and taxonomic interplay of proteins in microbiomes," should have been in the Table 1, or to reuse the Table 1 here.*

**We have highlighted this in table 1. We have kept the text here to provide the context for the rest of the sentence.**

*- (minor) The tenth paragraph of this section starts with the mention of multiple major challenges, but detail "only" one of them. I would suggest to mention some of the others challenges.*

*- (minor) The "MIA" method is just a hyperlink, without any reference. Either cite the website accordingly or add the reference.*

**There is not a reference, and the hyperlink goes to the GitHub site, as requested by the authors. We have included “ https://github.com/microbiome/mia” for completeness sake.**

*### Table 4*

*- (major) I was surprised to see that the objective "Foster international collaborations between other resources providers and databases to ensure global harmonisation of e-infrastructures for microbiome research" was long-term, as I would have imagined that a gap analysis would be short-term to ensure we do not reinvent the wheel, especially given the others initiatives discussed in the manuscript.*

*- (minor) If the Objective column starts with action verbs (which is a good idea), then it should be "Survey the needs" instead of "Survey of needs".*

**We have amended accordingly.**

*- (minor) Specify the type of workflow with "Address knowledge gaps in generating and adopting **data analysis** workflows"*

**Added**

*- (minor) The verb is missing in " **Teach a**dvanced containerisation and cloud deployment"*

**Added.**

*- (minor) The verb is missing in " **Promote** data analysis through the use of services". Is this ELIXIR services in general or specific ELIXIR Microbiome Community services?*

**Added Promote - generalised to ELIXIR services.**

*- (minor) Use "Share" instead of "Sharing" in the "Co-ordinate" entry.*

**Done**

*- (minor) The "Industry connection" entry does not fit the action verb pattern. A suggestion would be "Use ELIXIR and Node forums to understand pharmaceutical and biotechnological demands and current limitations impacting this sector." as the first sentence felt generic.*

**Done**

*- (minor) The verb is missing in " **Design** targeted training for different microbiome communities"*

**Done**

*- (minor) Use "findability" instead of "discoverability" for consistency and to fit with the FAIR principles.*

**Done**.

*- (minor) Reorder the sentence to start with the action verb: " **E**stablish new standards for microbiome research, particularly with respect to data analysis reporting and contextual metadata reporting **in conjunction with GSC**"*

**Done**

*- (minor) Correct "Established" to "Establish" in the "Promoting new approaches" entry.*

**Done**

*Editorial comments:*

*- (major) "Community" is used in upper-case and this is unclear in many instances whether the ELIXIR Marine Metagenomics Community is referred to, the ELIXIR Microbiome Community, or the broader microbiome research community. I suggest to use consistently the full term for the sake of transparency. Abbreviations like EMMC and EMC could be even more misleading in my opinion.*

**We have prefixed all instances of “Community” with their explicit community name.**

*- (major) The structure of the white paper is not evident as the hierarchy is indicated only by change in font size, and some sections are quite lengthy for a white paper that is supposed to be concise. I understand that this is a constraint from the Editor, but see Review reference 4 for a white paper with a more clearer structure. An alternative could be to use numbered sections.*

**We have introduced section numbers to aid the structuring of the manuscript.**

*- References*

*- (minor) The very first reference is oddly formatted in the text creating an artificial and confusing end of sentence.*

*- (minor) Title of reference 9 is truncated and should be "Methods included: standardizing computational reuse and portability with the Common Workflow Language"*

*- (minor) The superscript numbers of the numeric style of bibliography are in many instances after the final dot (see reference 2, 12-16, 17-19, 36, 37), after a comma (see reference 10, 20, 23), a bracket (see reference 60) or semi colon (see reference 65) when they should be before any of these symbols.*

*- (minor) In the paragraph "Interactions with other ELIXIR Communities", we jump from reference 24 to 29 when the numeric style of bibliography (that was chosen by the authors) is expected to mirror the mentions in the manuscript. Please either have the Table 2 earlier in the paper, or change the order of the references.*

**References have been fixed**
